# Dynamic RBM47 ISGylation confers broad immunoprotection against lung injury and tumorigenesis via TSC22D3 downregulation

**DOI:** 10.1038/s41420-023-01736-z

**Published:** 2023-11-30

**Authors:** Shihui Ding, Xiquan Pang, Shaoxiang Luo, Huili Gao, Bo Li, Junqiu Yue, Jian Chen, Sheng Hu, Zepeng Tu, Dong He, Youyi Kuang, Zhiqiang Dong, Min Zhang

**Affiliations:** 1 College of Biomedicine and Health, College of Life Science and Technology, Huazhong Agricultural University, Wuhan, 430070 China; 2 Center for Neurological Disease Research, Taihe Hospital, Hubei University of Medicine, Shiyan, 442000 Hubei China; 3 Wuhan Huamei Biotech Co., Ltd, Wuhan, 430206 China; 4 Department of Pathology, Hubei Cancer Hospital, Tongji Medical College, 430079 Wuhan, China; 5 Department of Head and Neck Surgery, Hubei Cancer Hospital, Tongji Medical College, 430079 Wuhan, China; 6 Department of Oncology, Hubei Cancer Hospital, Tongji Medical College, Wuhan, 430079 China; 7 Heilongjiang River Fisheries Research Institute of Chinese Academy of Fishery Sciences, No. 232, Hesong Street, Daoli District, Harbin, 150070 China

**Keywords:** Post-translational modifications, Lung cancer

## Abstract

ISGylation is a well-established antiviral mechanism, but its specific function in immune and tissue homeostasis regulation remains elusive. Here, we reveal that the RNA-binding protein RBM47 undergoes phosphorylation-dependent ISGylation at lysine 329 to regulate immune activation and maintain lung homeostasis. *K329R knockin* (KI) mice with defective RBM47-ISGylation display heightened susceptibility to LPS-induced acute lung injury and lung tumorigenesis, accompanied with multifaceted immunosuppression characterized by elevated pro-inflammatory factors, reduced IFNs/related chemokines, increased myeloid-derived suppressor cells, and impaired tertiary lymphoid structures. Mechanistically, RBM47-ISGylation regulation of the expression of TSC22D3 mRNA, a glucocorticoid-inducible transcription factor, partially accounts for the effects of RBM47-ISGylation deficiency due to its broad immunosuppressive activity. We further demonstrate the direct inhibitory effect of RBM47-ISGylation on TSC22D3 expression in human cells using a nanobody-targeted E3 ligase to induce site-specific ISGylation. Furthermore, epinephrine-induced S309 phosphorylation primes RBM47-ISGylation, with epinephrine treatment exacerbating dysregulated cytokine expression and ALI induction in *K329R KI* mice. Our findings provide mechanistic insights into the dynamic regulation of RBM47-ISGylation in supporting immune activation and maintaining lung homeostasis.

## Introduction

ISGylation is a ubiquitination-like post-translational modification involving the covalent linkage of interferon-stimulated gene 15 (ISG15) protein to the lysine residue of target proteins through the consecutive action of E1-activating enzyme (UBA7), E2-conjugating enzyme (UBE2L6), and E3 ligases (ARIH1, TRIM25, or hHERC5/mHERC6) [1, 2]. The process of de-ISGylation, which removes ISG15 from the target protein, is mediated by the enzyme ubiquitin-specific peptidase 18 (USP18) [3]. ISGylation is well known for its role in innate antiviral immunity induced by type I interferon [4, 5]. Recent studies have revealed that ISGylation is involved in a variety of cellular processes, such as DNA repair, autophagy, protein translation, and exosome secretion, and its dysregulation has been associated with human diseases, including cancer and neurodegenerative disorders [5–9]. ISG15 and ISGylation conjugating enzymes are frequently elevated in human malignant tumors, whereas some studies suggest a tumor-suppressive role of ISGylation in certain cancer cell lines, such as hepatocellular carcinoma and lung cancer [10–13]. Numerous proteins have been identified as substrates of ISG15 and have been linked to the regulation of diverse cellular processes involved in cancer progression [14–17]. Despite these findings, the precise physiological role of ISGylation in tissue protection remains incompletely elucidated.

RNA binding motif 47 (RBM47) is a novel RNA-binding protein (RBP) with diverse functions in different tissues by regulating mRNA stability, alternative splicing, miRNA biogenesis, and RNA editing [18–22]. Recent studies suggest that RBM47 acts as a tumor suppressor by stabilizing tumor suppressor mRNAs [23–26]. Additionally, RBM47 plays a crucial role in immune modulation by delaying IL-10 mRNA degradation in B cells [27], and negatively regulating IFN production during the innate antiviral immune response in fish [28].

Glucocorticoid-inducible transcriptional regulator TSC22 domain family member (TSC22D3), also known as glucocorticoid-induced leucine zipper, is a glucocorticoid-responsive molecule that possesses anti-inflammatory and immunosuppressive qualities [29, 30]. Interestingly, stress increases plasma glucocorticoids levels, which in turn induce the expression of TSC22D3, resulting in the blockade of the production of IFNs [29]. Moreover, macrophages activate TSC22D3 in response to IL-10 signaling, leading to the suppression of pro-inflammatory chemokines production [31]. Overall, ample studies have shown that TSC22D3 exerts broad immunosuppressive and anti-inflammatory effects [32–34], and overexpression of this molecule is associated with tissue damage and poor prognosis in cancer [35–37].

Recent research has identified RBM47 as a novel interferon stimulate gene (ISG) that exerts broad-spectrum antiviral activity and enhances host IFN downstream signaling by preserving the IFN-α/β receptors 1 (IFNAR1) mRNA [38]. Consistent with being an ISG, we found that RBM47 is ISGylated at lysine 329 (K329). To investigate its function, a knock-in (KI) mouse model was generated with an ISGylation-resistant mutation (K329R). Further investigations revealed that deficiency of RBM47-ISGylation (K329R KI) leads to multifaceted immunosuppression by inducing TSC22D3 expression, which impairs tissue homeostasis, leading to acute lung injury (ALI) and lung tumorigenesis.

## Results

### RBM47 is ISGylated at Lys329

**Fig. 1:**
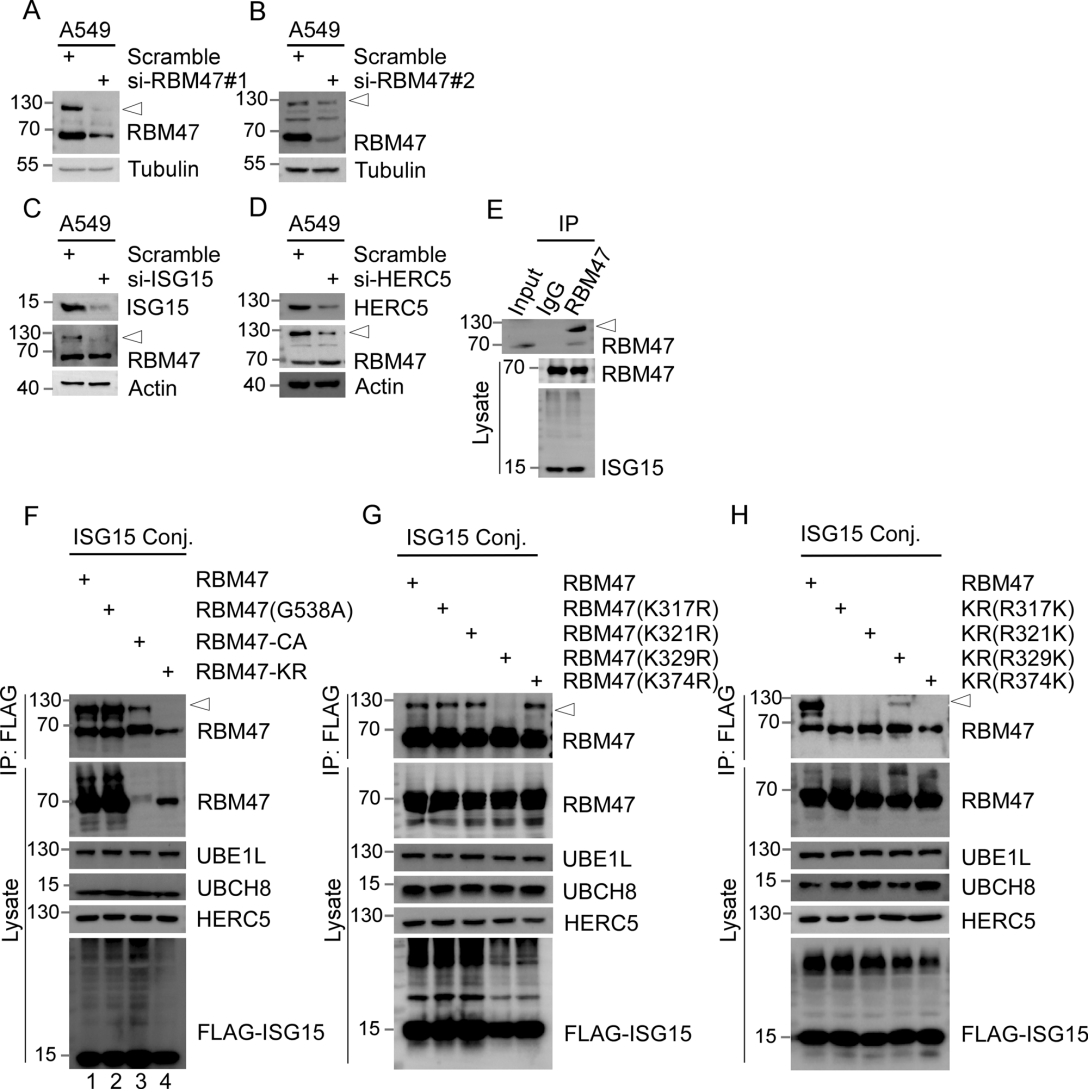
RBM47 is ISGylated at Lys329. Endogenous RBM47 showed a slower-migrating band (indicated by an arrowhead), which was reduced upon the knockdown of RBM47. RBM47 siRNA#1 (**A**) and RBM47 siRNA#2 (**B**), and scrambled siRNA were transfected into A549 cells for 72 h, followed by WB analysis (WB) using antibodies recognized RBM47 and Tubulin. Depletion of ISG15 (**C**) and HERC5 (**D**) inhibited ISGylation of RBM47. The slower migrating band of RBM47 (arrowhead) was reduced upon the knockdown of ISG15 or HERC5. ISG15 siRNA (**C**), HERC5 siRNA (**D**), and scrambled siRNA were transfected into A549 cells for 72 h, followed by WB analysis using antibodies recognized ISG15, HERC5, Actin, and RBM47. **E** Endogenous RBM47 is ISGylated. 293 T cells were co-transfected with plasmids expressing components of the ISGylation conjugation system (ISG15 conj.), including UBE1L, UBCH8, HERC5, and FLAG-ISG15. 48 h after transfection, cell lysates were subjected to immunoprecipitation (IP) using anti-FLAG magnetic beads followed by WB analysis using antibody-recognized RBM47 (upper panel). And the cell lysates were analyzed by WB using antibodies recognized RBM47 and ISG15 (lower panel). **F** The ISGylation of RBM47 was abolished by mutating all lysines to arginine (RBM47-KR mutant). 293 T cells were transfected with plasmids expressing ISGylation modification enzymes as in (**E**), plus plasmids expressing wild-type RBM47 (line 1) and RBM47-KR mutant (line 4) as indicated. In addition, G538A mutant (line 2) and RBM47-CA (all cysteine to arginine) mutant (lane 3) were included as controls. 48 h after transfection, cell lysates were subjected to IP using anti-FLAG magnetic beads, followed by WB analysis using antibody recognized RBM47. And the cell lysates were analyzed by WB using antibodies as indicated. **G** The ISGylation of RBM47 was reduced by mutation of lysine 329 to arginine (K329R). The experiments were down as in (**F**), except that RBM47 and a series of RBM47 lysine (K) to arginine (R) substitution mutants (including K317R, K321R, K329R, and K374R) were expressed. **H** K329 is the acceptor site for ISGylation. In the RBM47-KR mutant, each arginine mutation was reversed back to lysine one at a time. The experiments were down as in (**F**), except that RBM47 and KR mutant with R to K back mutation (including R317K, R321K, R329K, and R374K) were expressed.

We investigated RBM47 expression across various cell lines, including the human cancer cell line A549 (Fig. S1A). Our Western blot (WB) assay with antibodies raised against amino acids 565–580 of RBM47 revealed a slower-migrating band of RBM47 (arrowheads) with a molecular mass of ~130 kDa (Fig. 1A, B). The use of two different siRNAs to knockdown RBM47 resulted in a significant decrease of this specific band (arrowheads) as well as the overall expression of RBM47 in A549 cells (Fig. 1A, B). We postulated that post-translational modifications could be responsible for the reduced mobility of RBM47. To investigate this, we expressed FLAG-RBM47 in HEK293T cells, followed by anti-FLAG immunoprecipitation (IP) combined with mass spectrometry. Our findings suggest that RBM47 may interact with many proteins from the Ubiquitin-Proteasome System. BioGRID database indicates a potential interaction between RBM47 and HERC5 [39] (Fig. S1B). HERC5 is a major E3 ligase for ISG15 conjugation. We therefore hypothesized that ISGylation of RBM47 could result in slower migration of the protein. The lipopolysaccharide (LPS) of bacterial cell walls can mimic bacteria and activate macrophages, thereby triggering an inflammatory response. Treatment with LPS results in an elevated level of ISG15 conjugates in macrophages [40]. As expected, LPS induced the slower migrating form of RBM47 (arrowheads) in a time- and dose-dependent manner in A549 cells (Fig. S1C, D). More importantly, the knockdown of components of the ISGylation conjugation system, such as ISG15, UBE1L (E1), UBCH8 (E2), and HERC5 (E3), inhibited the slower migrating form of RBM47 (Figs. 1C, D and S1E, F).

ISGylation events can be verified by building an artificial ISGylation conjugation system. In order to construct a system incorporating the necessary components for ISGylation events, we employed molecular cloning techniques to construct five plasmids: ISG15 protein, E1, E2, E3, and the target protein. These five plasmids were co-transfected into cells. This co-expression facilitates the activation, transfer, and attachment of ISG15 to the target protein, thereby simulating ISGylation events. The success of ISGylation events is validated using Western blotting, allowing for the assessment of the ISGylation effect [41]. Accordingly, we co-expressed FLAG-ISG15 with UBE1L, UBCH8, and HERC5 in 293 T cells, followed by IP with anti-FLAG antibodies. Immunoblotting of anti-FLAG immunoprecipitates revealed that ISG15 was conjugated to endogenous RBM47, as demonstrated by a shifted band that consistent with the slower migrating form of RBM47 (Fig. 1E). ISGylation occurs on lysine (K) residues, whereas arginine (R) has similar properties to lysine but cannot be ISGylated. We constructed RBM47-KR mutant with all lysine-to-arginine mutations, which abolished the ISGylation of RBM47 in the presence of the ISGylation conjugation system, compared to wild-type RBM47 (Fig. 1F). In other studies, we generated RBM47-CA mutant with all cysteine-to-alanine mutations and a clinically relevant G538A mutant, both of which were still subjected to ISGylation (Fig. 1F). In addition, overexpression of USP18, a ubiquitin-like carboxy-terminal hydrolase, reduced the slower migrating form of RBM47 in the presence of the ISGylation conjugation system (Fig. S1G). These data suggest that RBM47 is subject to ISGylation, leading to a distinctive alteration in its banding pattern.

Then, we constructed a series of mutants containing K to R mutation at individual lysine site to identify the acceptor site for RBM47 ISGylation. Among them, K329R obviously reduced the ISGylation of RBM47 (Fig. 1G). Accordingly, reintroduction of K329 in RBM47-KR mutant effectively restored the ISGylation of RBM47 (Fig. 1H). Taken together, we identified that RBM47 is a target of ISGylation with K329 as its acceptor site.

### Deficiency of RBM47-ISGylation induces multifaceted immunosuppression in the lung

The distribution of lysine in mouse RBM47 is identical to that of human RBM47, except for the presence of an additional K332 residue (Fig. S2A). Upon exposure to the ISGylation conjugation system, two ISGylated bands appeared in mouse RBM47, with K329 accounting for the lower band (Fig. S2B). K332 of mouse RBM47 accounts for the upper band (Fig. S2B). In contrast, the corresponding residue in human RBM47 is an R332 residue (Fig. S2A). To address this discrepancy and generate a mouse model that is consistent with human RBM47, we developed *K329R; K332R* double knock-in (KI) mice on a C57BL/6 background. This strategy not only eliminates the risk of role redundancy associated with the additional K332 residue, but also ensures similarity to the human form of RBM47 (Fig. S2C). For simplification purposes, *K329R; K332R* was referred to as *K329R* in this study. Notably, *K329R KI* mice were developmentally normal and fertile.

**Fig. 2:**
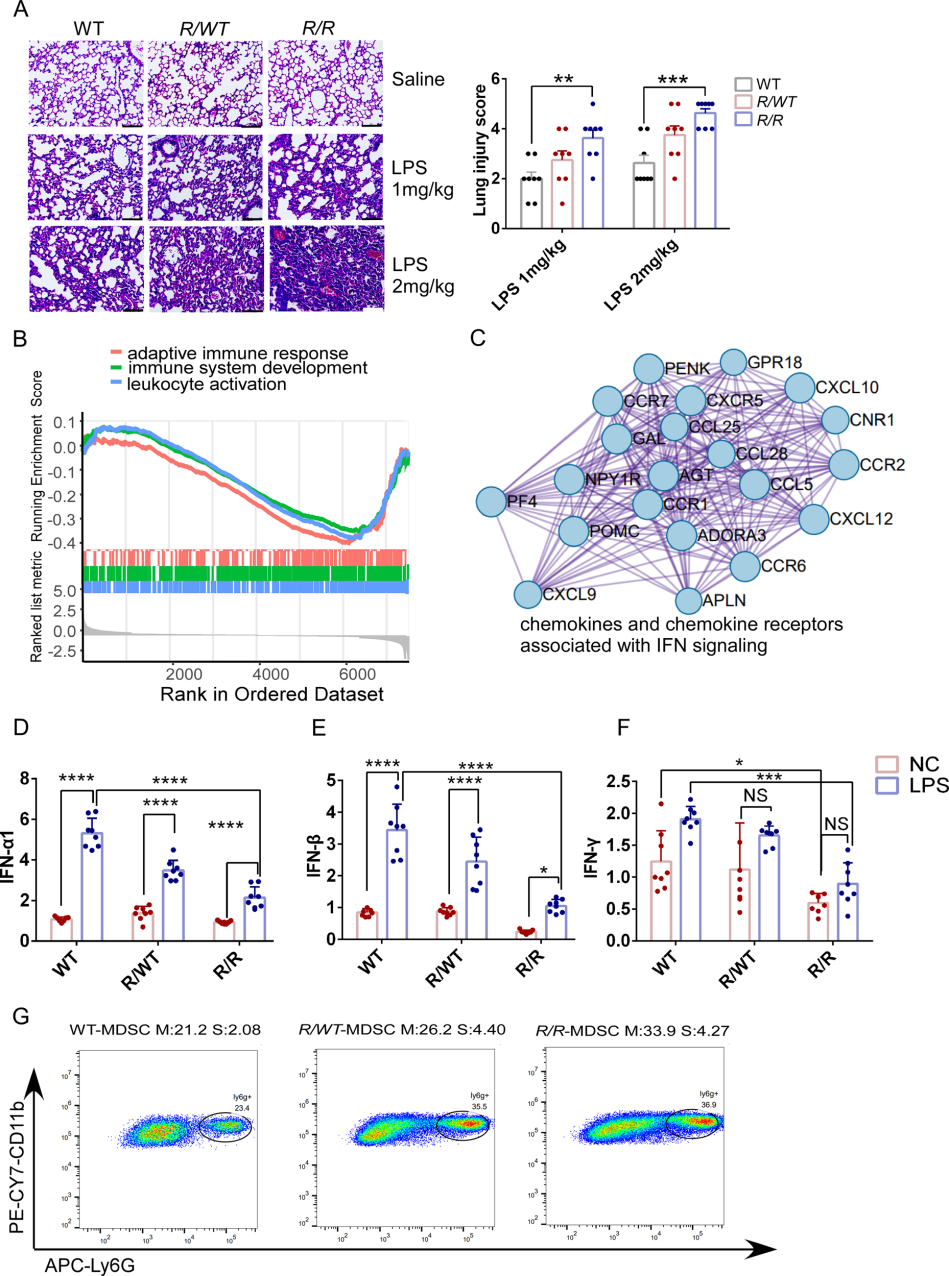
Deficiency of RBM47-ISGylation induces multifaceted immunosuppression in the lung. **A** K329R KI exaggerated LPS-induced acute lung injury (ALI). Mice were injected intraperitoneally with LPS (1 mg/kg or 2 mg/kg) or vehicle (Saline). 24 h later, lung tissues were analyzed by H&E. Representative images of H&E-stained lung tissue sections (*n* = 8), Scale bars, 75 μm (Left panel). Statistical graph of lung injury scores based on histological analysis of the lung tissues. Data are presented as the mean ± SEM (*n* = 8). Statistical significance was determined by two-way ANOVA (*n* = 8 for each genotype; *, *p* value < 0.05, **, *p* value < 0.01, ***, *p* value < 0.001, ****, *P* < 0.0001) (Right panel). **B** Based on Gene Set Enrichment Analysis (GSEA) with ClusterProfiler, it appears that genes which are dysregulated in R/R mice are primarily associated with immune system-related processes including adaptive immune responses, immune system development, and leukocyte activation. **C** PPI network and MCODE analysis of DEGs. MCODE algorithm was applied to identify densely connected network components. Chemokines and chemokine receptors in MCODE1 were enriched in IFN signaling. The PPI network was built and visualized by Cytoscape v3.6.0. Repression of basal and LPS-induced IFNs in mice. Mice were injected intraperitoneally with LPS 2 mg/kg, and 24 h later, the mRNA expression levels of *IFN-α1* (**D**), *IFN-β* (**E**), and *IFN-γ* (**F**) were examined by real-time quantitative reverse transcription PCR (RT-qPCR) on lung RNA, isolated from *R/R*, *R/WT*, and WT mice. All samples were normalized using GAPDH housekeeping gene expression. Data were presented as mean ± SEM. Statistical significance was determined by two-way mean ANOVA (*n* = 8 for each genotype; *, *p* value < 0.05, **, *p* value < 0.01, ***, *p* value < 0.001, ****, *P* < 0.0001). **G** Gating strategy for identification of MDSC (CD11b+, Ly6G+). Frequency of MDSC in the lungs of WT, *R/R,* and *R/WT* mice after LPS administration (*n* = 5). 24 h after injection of LPS (2 mg/kg), lung tissue’s myeloid-derived suppressor cells (MDSCs) were identified by flow cytometry. Data were acquired using a cytoflex-LX flow cytometer. Compensation was performed on the cytoflex-LX flow cytometer at the beginning of each experiment. Data were analyzed with Flowjo v10. M Mean and S SEM.

To induce ISGylation in mice, we employed injection of LPS. Interestingly, we observed reduced expression of basal RBM47-ISGylation in the lungs of heterozygous K329R (*R/WT*) and homozygous *R/R* mice compared to wild-type WT mice (Fig. S2D). Furthermore, LPS treatment induced RBM47-ISGylation in the lungs of WT and *R/WT* mice, but not in *R/R* mice (Fig. S2D). Histological analysis revealed that LPS-induced acute lung injury (ALI) was evident in all three genotyped mice, as demonstrated by disorganized lung architecture, alveolar septa thickening, and inflammatory cell infiltration (Fig. 2A, Left panel, *n* = 8 for each genotype). The severity of ALI was assessed using a semiquantitative histopathology score system (Table S3), and remarkably, *R/WT* and *R/R* mice demonstrated more severe ALI than WT controls, with *R/R* mice experiencing the most severe ALI, underscoring the critical role of RBM47-ISGylation in the lung (Fig. 2A, Right panel). Furthermore, the non-invasive approach of nebulized-LPS resulted in similar findings, with *R/WT* and *R/R* mice showing more severe ALI than WT mice (Fig. S2E).

To further investigate the molecular mechanisms underlying the observed ALI phenotype, we performed bulk RNA-seq on the lungs of *R/R* mice and WT littermates (8 weeks of age). Gene Set Enrichment Analysis (GSEA) indicated that differentially expressed genes (DEGs) were enriched in immune-related processes, including adaptive immune response, immune system development, and leukocyte activation (Fig. 2B). Using Metascape analysis, we constructed a protein-protein interaction (PPI) network of DEGs, which identified six significant MCODE modules. Notably, MCODE 1 was highly related to IFN signaling and consisted of 22 genes abundant in chemokines and chemokine receptors associated with IFN signaling (Fig. 2C). These chemokines and chemokine receptors are known to orchestrate a wide array of leukocyte functions during inflammation and immunity [42]. To validate these findings, we used real-time quantitative reverse transcriptase PCR analysis (RT-qPCR) to assess the expression levels of chemokines and chemokine receptors in the lungs of the three genotyped mice. We found that the basal mRNA expression of *CCR2*, *CCL5, CXCL9*, and *CXCL10* in lungs were decreased in *R/R* mice compared with those in WT mice (Fig. S2F–I). The mRNA expression of these genes was up-regulated in the lungs upon LPS stimulation. However, LPS-induced mRNA expression of these genes was repressed in *R/R* mice (Fig. S2,F-I). The basal and LPS-induced mRNA expression of these genes in *R/WT* mice were also relatively lower than those in WT mice (Fig. S2F-I). Additionally, we evaluated the baseline and LPS-induced mRNA expression levels of pro-inflammatory cytokine genes such as *TNF-α*, *IL-6*, and *IL-10* in the lungs of the three genotyped mice, and observed that the baseline and LPS-induced mRNA expression of *TNF-α* and *IL-10* remained consistent across all three genotyped mice. However, the expression of *IL-6* was notably upregulated in LPS-induced *R/R* mice relative to WT mice, whereas no significant difference was detected under normal saline treatment conditions (Fig. S2J-L).

Given the importance of IFN signaling in regulating chemokines and cytokines, we further assessed the mRNA expression levels of IFNs in the lungs of the three genotyped mice using RT-qPCR. Interestingly, we found that the basal mRNA expression level of *IFN-α1* and *IFN-β* in *R/R* mice were comparable to those in WT mice, while the basal mRNA levels of *IFN-γ* were lower in *R/R* mice than in WT mice (Fig. 2D–F). LPS stimulation significantly induced the mRNA expression of *IFN-α1*, *IFN-β*, and *IFN-γ* in WT mice, but this response was repressed in *R/R* mice compared to WT controls (Fig. 2D–F). Impressively, LPS hardly induced the mRNA expression of *IFN-γ* in *R/R* and *R/WT* mice (Fig. 2D–F). The basal mRNA expression level of *IFN-α1*, *IFN-β*, and *IFN-γ* in *R/WT* mice were comparable to those in WT mice (Fig. 2D–F). However, LPS-induced mRNA expression of *IFN-α1*, *IFN-β*, and *IFN-γ* were relatively repressed in *R/WT* mice, compared to in WT mice (Fig. 2D–F). Since RBM47-ISGylation most significantly regulates *IFN-γ*, we further investigated their relationship and found that intraperitoneal injection of *IFN-γ* increased the expression of RBM47-ISGylation in a dose-dependent manner in mice (Fig. S2M). Consistently, knockdown of *IFN-γ* decreased LPS-induced ISGylation of RBM47 in A549 cells (Fig. S2N).

To further investigate the immunological changes associated with the observed ALI phenotype, perfused lungs were collected to examine changes in the following immune cell populations using flow cytometry after LPS administration: CD4^+^ T, CD8^+^ T, macrophages, MDSC, and neutrophils. Interestingly, we observed a significant increase in the percentage of myeloid-derived suppressor cells (MDSCs, CD11b^+^ Ly6G^+^) in the lungs of *R/R* mice compared to WT mice (Fig. 2G). *R/WT* mice showed a relative increase in MDSCs compared to WT controls (Fig. 2G). MDSCs are a heterogeneous population of immature myeloid cells widely implicated in immune suppression and have been associated with the development of cancer [43–45]. Previous studies have shown that inflammatory cytokines CCL2 and GM-CSF promoted the expansion of MDSCs and their function. [43–45] Consistent to previous findings [46, 47], our results showed that LPS induced the mRNA expression of *CCL2*, *GM-CSF* in WT mice. Notably, LPS-induced *CCL2*, *GM-CSF* expression was further increased in *R/R* mice (Fig. S2O, P). Our results suggest that the deregulated secretion of pro-inflammatory factors such as *IL-6*, *CCL2*, and *GM-CSF*, along with the downregulation of immune factors such as *IFNs*, *CCL5*, *CXCL9*, and *CXCL10*, contribute to the multifaced immunosuppression observed in the lungs of *R/R* mice. Furthermore, the expansion of MDSCs in the lungs of *R/R* mice following LPS administration may play a significant role in the development of ALI.

### Deficiency of RBM47-ISGylation promotes lung tumorigenesis

The deficiency of RBM47-ISGylation may play a critical role in lung tumorigenesis due to pulmonary immunosuppression. To investigate this potential, we injected Lewis lung carcinoma cells (LLCs, 2.5 × 10^5^ cells) into the tail veins of C57BL/6 mice (*n* = 6; age, 8 weeks). The results showed an increased incidence of lung adenocarcinoma in *R/R* mice (83%) and *R/WT* mice (50%), compared to 33.3% in WT mice (Fig. S3A, B). In addition, the tumors’ weight increased in *R/R* mice than those in WT mice (Fig. S3C). Notably, Lewis lung carcinoma is a spontaneous adenocarcinoma of the lung originating in C57BL/6 mice [48], suggesting a potential link between RBM47-ISGylation deficiency and lung tumorigenesis in mice.

**Fig. 3:**
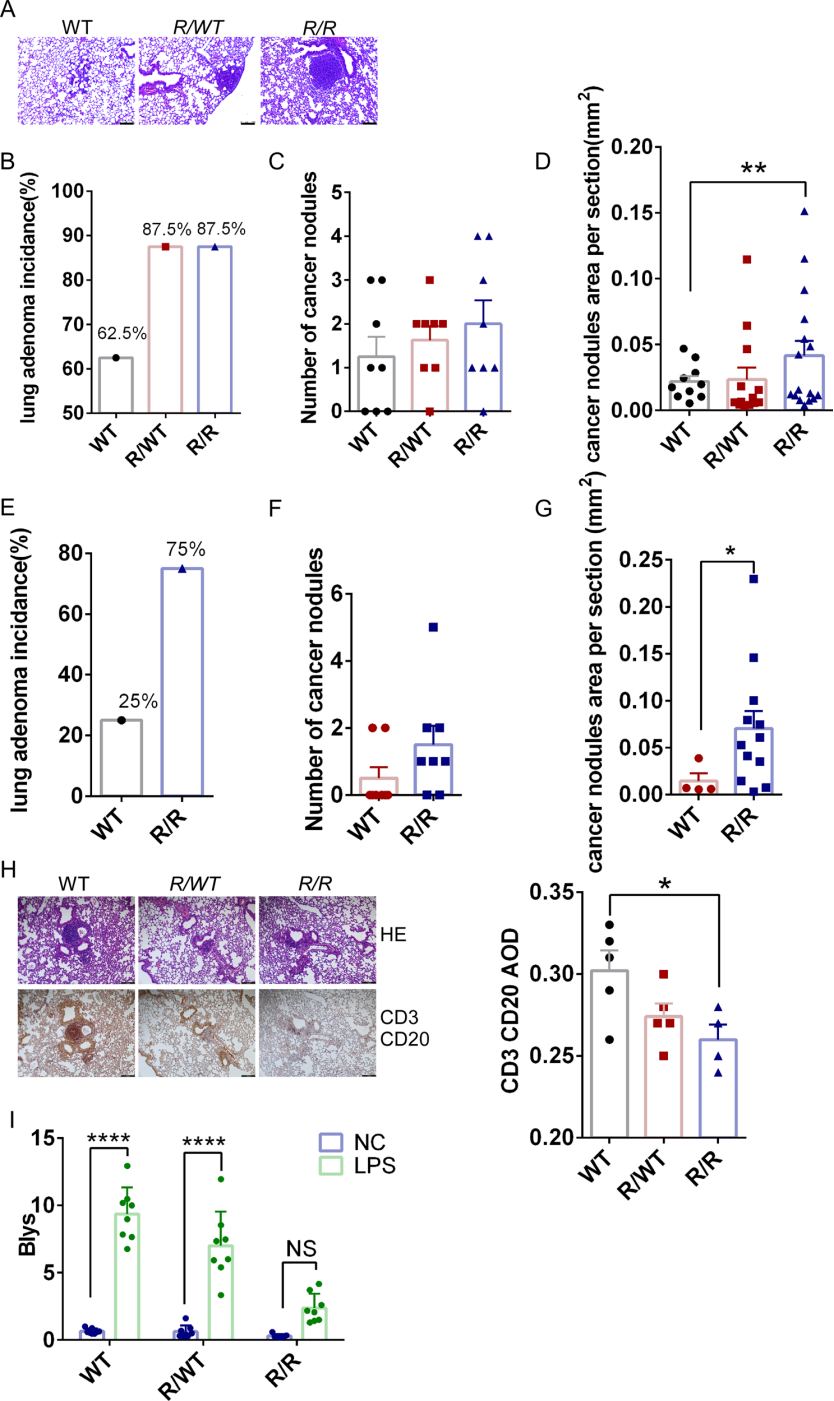
Deficiency of RBM47-ISGylation promotes lung tumorigenesis. **A** Representative images of lung cancer nodules in H&E-stained lungs of urethane-treated mice. *R/R*, *R/WT*, and WT mice (*n* = 8 for each genotype) received consecutive 10 weekly intraperitoneal urethane injections (1 g/kg, once a week), and 12 weeks after urethane injection, lung tissues were collected for H&E staining. Scale bars = 100 μm. **B** Statistical graph of the lung adenoma incidence in mice from the experiment (**A**). The number of lung cancer nodules (**C**) and sizes (**D**) observed in mice from the experiment (**A**). Data were shown as mean ± SEM. Statistical significance was determined by one-way mean ANOVA (*, *p* value < 0.05, **, *p* value < 0.01, ***, *p* value < 0.001). **E** Statistical graph of the lung adenoma incidence in mice. *R/R* and WT mice (*n* = 8 for each genotype) received consecutive 10 weekly intraperitoneal urethane injections (1 g/kg), and 8 weeks after urethane injection, lung tissues were collected to evaluate the incidence of lung adenoma. The number of lung cancer nodules (**F**) and sizes (**G**) observed in mice from the experiment (**E**). Data were shown as mean ± SEM. Statistical significance was determined by T-test (*, *p* value < 0.05, **, *p* value < 0.01, ***, *p* value < 0.001). **H** *K329R* KI prevents the formation of TLS (tertiary lymphoid structures). Immunohistochemical staining of serial sections of lung tissue from the mice shown in (**A**). Representative images of immunohistochemical staining for CD3 (for T lymphocytes, brown) and CD20 (for B lymphocytes, red) were shown (Left panel). Scale bar, 100 μm. Statistical analysis of CD3+ and CD20+ immunohistochemistry was performed by counting the average optical density (AOD) (*n* = 5). Statistical significance was determined by one-way mean ANOVA (*, *p* value < 0.05, **, *p* value < 0.01, ***, *p* value < 0.001) (Right panel). **I** Mice were injected intraperitoneally with LPS 2 mg/kg, and 24 h later, the mRNA expression levels of *Blys* were examined by real-time quantitative reverse transcription PCR (RT-qPCR) on lung RNA, isolated from *R/R*, *R/WT*, and WT mice. All samples were normalized using GAPDH housekeeping gene expression. Data are presented as mean ± SEM. Statistical significance was determined by two-way mean ANOVA (*n* = 8 for each genotype; *, *p* value < 0.05, **, *p* value < 0.01, ***, *p* value < 0.001, ****, *P* < 0.0001).

To further investigate the potential link between RBM47-ISGylation deficiency and lung tumorigenesis, we utilized the urethane-induced lung adenocarcinoma mouse model, which causes tumors primarily through Kras mutations [49]. *K329R KI* mice (*n* = 8; age, 8 weeks) and WT controls received weekly intraperitoneal injections of urethane (1 g/kg) for 10 weeks. Histological analysis conducted 12 weeks after urethane injection demonstrated infiltrative tumor growth with clear tumor borders in all three genotyped mice (Fig. 3A). The incidence of lung tumors was significantly higher in *R/R* mice (87.5%) and *R/WT* mice (87.5%) compared to WT mice (62.5%) (Fig. 3B). Moreover, a more significant tumor cell proliferation was observed in sporadic small adenoma areas of *R/R* mice, as revealed by higher tumor burden with increased size of cancer nodules (Fig. 3C, D). More importantly, lung adenomas were already present in *R/R* mice with an incidence of 75% (6 out of *n* = 8 mice) as early as 8 weeks after urethane administration (Fig. 3E, S3D). In contrast, WT mice developed lung adenomas with a much lower incidence of 25% (2 out of *n* = 8 mice) at this early stage (Fig. 3E). Additionally, a significant tumor cell proliferation was observed in sporadic small adenoma areas of *R/R* mice compared to WT controls, as revealed by higher tumor burden with increased size of cancer nodules (Fig. 3F, G). Tertiary lymphoid structures (TLS), which are ectopic lymphoid aggregates that help shape a favorable immune microenvironment to control tumor development, were also assessed [50]. Clusters of B (CD20^+^) and T lymphocytes (CD3^+^) can form TLS at the site of inflammation. Interestingly, immunohistochemical stains of CD3 and CD20 showed the establishment of TLS in WT mice, which was impaired in *R/WT* and *R/R* mice (Fig. 3H). B lymphocyte stimulator BLyS (also known as B cell activating factor, BAFF) is a cytokine involved in the development of B-cells, and reducing BLyS prevented the formation of TLSs [51]. Consistently, LPS-induced BLyS mRNA expression was repressed in *R/R* and *R/WT* lungs but not in WT lungs (Fig. 3I). These findings suggest that RBM47-ISGylation deficiency is linked to increased tumorigenesis in the lungs, likely through impaired immune surveillance in tumor microenvironment.

### Deficiency of RBM47-ISGylation leads to immunosuppression by up-regulating TSC22D3 mRNA expression

**Fig. 4:**
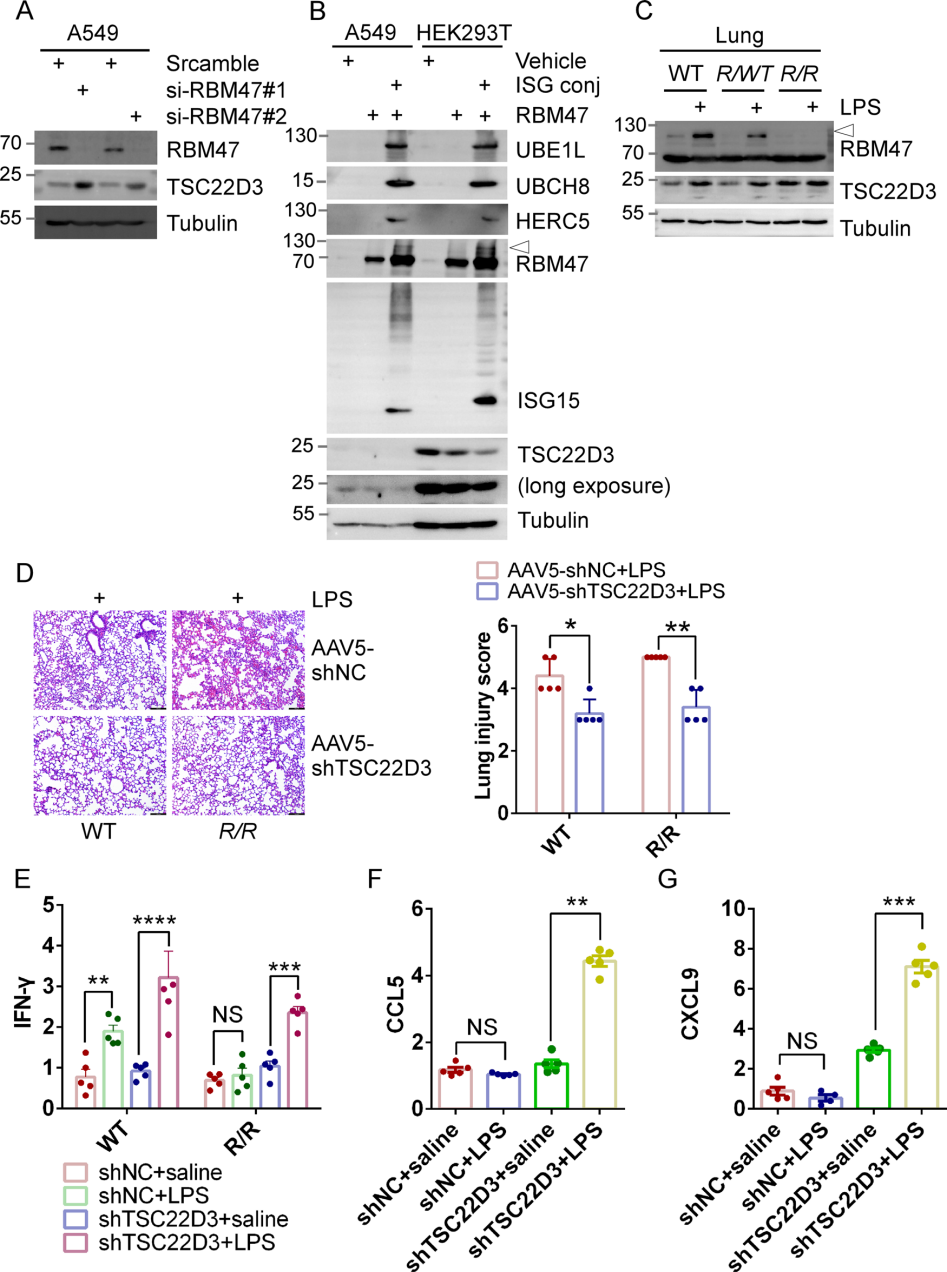
Deficiency of RBM47-ISGylation leads to immunosuppression by up-regulating TSC22D3 mRNA expression. **A** Knockdown of RBM47 increased the protein expression of TSC22D3. Two different siRNA against RBM47 and scrambled siRNA were transfected into A549 cells for 72 h, followed by WB analysis using antibodies recognized RBM47, TSC22D3, and Tubulin. **B** A549 and 293 T cells were mock-transfected or transfected with pcDNA3.1 plasmid expressing RBM47 for 48 h, co-transfected with ISG15, UBE1L, UBCH8, HERC5 (ISG15 Conj.) as indicated, followed by WB analysis using antibodies as indicated. **C** Deficiency of RBM47-ISGylation increased TSC22D3 expression in the lungs of *R/R* mice. LPS (2 mg/kg) were intraperitoneally injected into *R/R*, *R/WT*, and WT Mice, and 24 h later lung tissues were collected, followed by WB analysis using antibodies recognized RBM47, TSC22D3, and Tubulin. **D** Knockdown of TSC22D3 ameliorated LPS-induced acute lung injury (ALI) in *R/R* and WT mice. *R/R* and WT mice were received intratracheal injection of AAV5-TSC22D3 and control AAV5-shNC (2×10^11^ vg) for 6 weeks, followed by LPS injection (2 mg/kg). Histological analysis of lung sections stained with H&E (*n* = 5 per group), Scale bar, 100 μm (Left panel). Statistical graph of lung injury scores based on histological analysis of the lung tissues. Data are presented as the mean ± SEM (*n* = 5). Statistical significance was determined by two-way ANOVA (*n* = 5 for each genotype; *, *p* value < 0.05, **, *p* value < 0.01, ***, *p* value < 0.001, ****, *P* < 0.0001) (Right panel). **E** Knockdown of TSC22D3 recovered LPS-induced mRNA expression of *IFN-γ*. *R/R* and WT mice received intratracheal injection of AAV5-TSC22D3 and control AAV5-shNC (2 × 10^11^ vg) for 6 weeks, followed by saline or LPS injection (2 mg/kg). mRNA expression of *IFN-γ* in the lungs was analyzed by real-time quantitative reverse transcription PCR (RT-qPCR) and normalized to GAPDH levels. Data were presented as mean ± SEM. Statistical significance was determined by two-way mean ANOVA (*n* = 5 *, *p* value < 0.05, **, *p* value < 0.01). Knockdown of TSC22D3 recovered LPS-induced mRNA expression of *CCL5* and *CXCL9*. The experiments were done as in (**E**), except that mRNA expression of *CCL5* (**F**), and *CXCL9* (**G**) in *R/R* lungs was analyzed.

To investigate the molecular mechanisms underlying RBM47-ISGylation deficiency-mediated immunosuppression, we screened key genes involved in immunosuppression using RNA-seq data obtained from our study and a published RNA-seq dataset (GSE75491) from NSCLC cells in which RBM47 was knocked down [52]. Interestingly, we identified the master immunosuppressive gene TSC22D3 may be a key mediator of RBM47-ISGylation deficiency-induced immunosuppression. To validate these findings, knockdown of RBM47 increased the mRNA expression of TSC22D3, while overexpression of RBM47 decreased TSC22D3 expression in A549 cells (Fig. S4A, B). Additionally, knockdown of RBM47 increased the protein expression of TSC22D3 in A549 cells (Fig. 4A). Further experiments using RNA immunoprecipitation (RIP) and electrophoretic mobility shift assay (EMSA) showed that RBM47 bound with the mRNA of TSC22D3 (Fig. S4C, D). Moreover, overexpression of RBM47 more effectively inhibited TSC22D3 expression in A549 and 293 T cells in the presence of the ISGylation conjugation system compared with the absence of the ISGylation conjugation system (Fig. 4B).

Importantly, our study provides evidence that RBM47-ISGylation deficiency induces immunosuppression by upregulating the expression of TSC22D3 in the lung. Indeed, we observed an increased protein expression level of TSC22D3 in *R/R* lungs, along with decreased expression of RBM47-ISGylation, as compared to the lungs of littermate WT controls (Fig. 4C). Notably, LPS stimulation increased the expression of TSC22D3 in WT and *R/WT* lungs, but not in *R/R* lungs (Fig. 4C). Immunohistochemical (IHC) staining also showed the highest basal expression of TSC22D3 in *R/R* lungs compared to that in WT lungs (Fig. S4E). Consistently, expression of TSC22D3 was significantly increased in lung tissues of urethane-treated *R/R* mice described above, compared to littermates WT mice (Fig. S4F). Although it is currently challenging to isolate sufficient specific immune cells from lung tissue for TSC22D3 expression analysis, we isolated tumors and their adjacent tissues (<0.2 cm from the tumor margin) from the Lewis lung carcinoma xenograft model described above and observed significantly increased TSC22D3 expression in xenografts of *R/R* mice compared with WT (Fig. S4G).

To further investigate the potential role of TSC22D3 in mediating the immunosuppressive effects of RBM47-ISGylation deficiency, we used adeno-associated virus (AAV5) to knock down TSC22D3 expression in lungs of *R/R* and littermate WT mice. Injection of AAV5-shTSC22D3 for 6 weeks resulted in decreased basal and LPS-induced expression of TSC22D3 in lungs, as compared to AAV-shNC controls (Fig. S4H, I). IHC analysis also showed decreased LPS-induced expression of TSC22D3 in the lungs of AAV5-shTSC22D3-treated mice, compared to AAV-shNC controls (Fig. S4J). Consequently, knockdown of TSC22D3 alleviated the exacerbation of ALI observed in *R/R* mice (Fig. 4D). Consistently, knockdown of TSC22D3 reconstituted LPS-induced *IFN-γ* expression in *R/R* mice but not in WT mice (Fig. 4E). Furthermore, knockdown of TSC22D3 recovered LPS-induced *CCL5* And *CCXL9* in *R/R* mice (Fig. 4F, G). But, knockdown of TSC22D3 did not affect the mRNA expression of IL-6. These results provide compelling evidence that TSC22D3 plays a critical role in mediating the immunosuppressive effects of RBM47-ISGylation deficiency in the lung.

### Chimeric E3 ligase induces human RBM47-ISGylation that represses TSC22D3 expression

To confirm the role of RBM47-ISGylation, it would be valuable to induce its ISGylation specifically. E3 ligase plays a critical role in transferring ubiquitin or ubiquitin-like proteins such as ISG15 from the E2-conjugating enzyme to the lysine site of targeted substrates. The specificity of E3 ligase in selecting substrates can be modulated by utilizing intrabodies that bind to specific targets, replacing its natural substrate-binding domain [53]. Building upon this concept, a chimeric E3 ligase can be designed by incorporating a nanobody to replace the substrate-binding domain, thus enabling site-specific ISGylation induction.

Chimeric E3 ligases have emerged as a powerful tool for targeted protein degradation through the bioPROTAC strategy [54]. In this study, we successfully generated an anti-RBM47 nanobody (nbRBM47) using a 12 amino acid peptide spanning the ISGylation site (CKEQYSRYQKAA) as an epitope to screen yeast surface display libraries of synthesis nanobody. In line with the bioPROTAC strategy, nbRBM47 was fused to a truncated adaptor protein SPOP, resulting in the nbRBM47-SPOP chimera (Fig. S5A). A general negative control nanobody bv025 was used to construct bv025-SPOP [55]. Results showed that the chimeric nbRBM47-SPOP ligase effectively decreased the protein expression of RBM47-mCherry and endogenous RBM47 in 293 T cells, compared with bv025-SPOP (Fig. S5B). Notably, nbRBM47-SPOP ligase decreased the protein expression of RBM47 in A549 cells, resulting in increased expression of TSC22D3 (Fig. S5C).

**Fig. 5:**
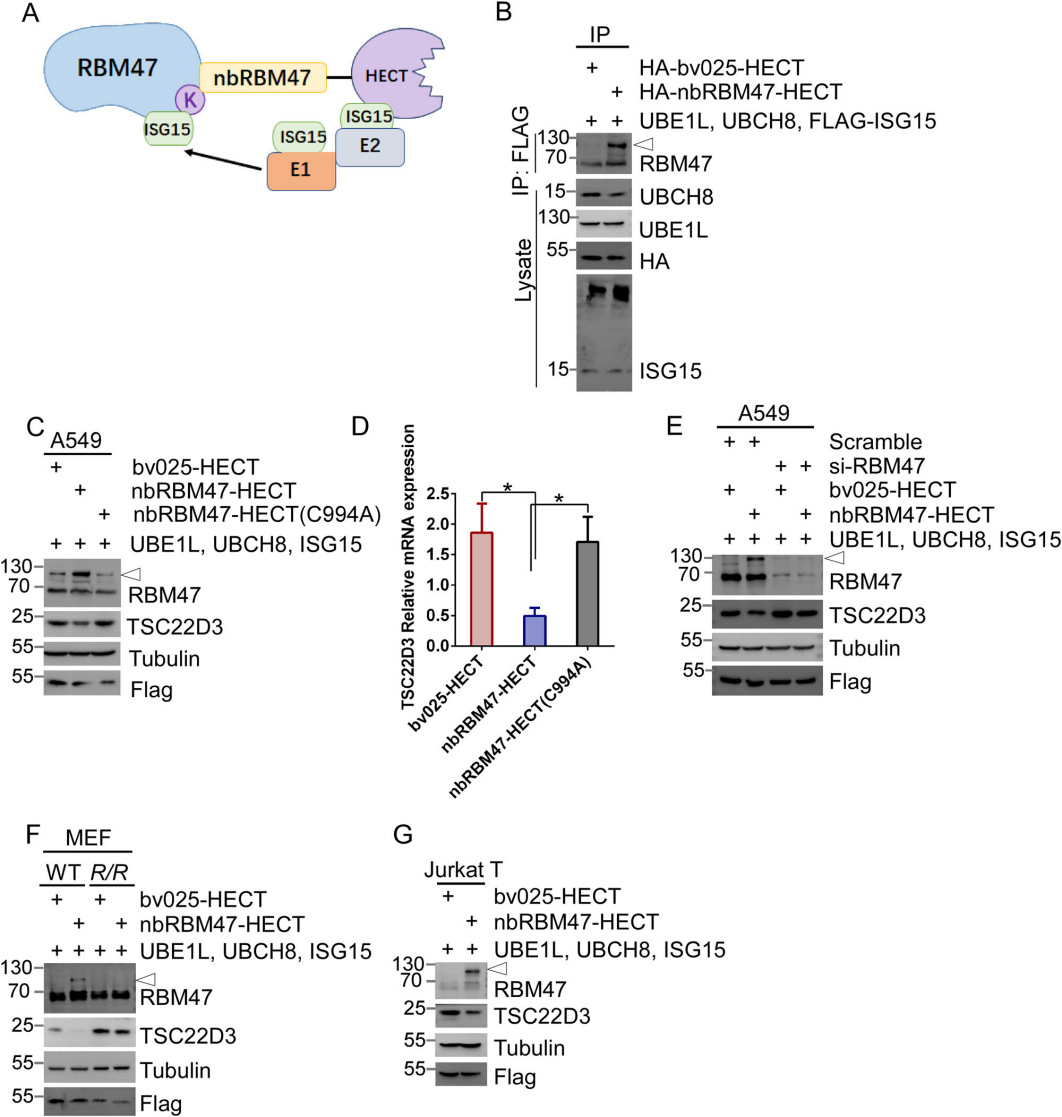
Chimeric E3 ligase induces human RBM47-ISGylation that represses TSC22D3 expression. **A** Design of chimeric E3 ligase (nbRBM47-HECT) by fusing nbRBM47 with HERC5_681–1024_ (HECT domain). The substrate recognition domain of the E3 adaptor HERC5 was replaced by nbRBM47. Negative nanobody bv025 was used as a control to construct bv025-HECT. **B** nbRBM47 induced ISGylation of RBM47 in 293 T cells. 293 T cells were co-transfected with plasmids expressing UBE1L, UBCH8, and FLAG-ISG15, plus HA tagged nbRBM47-HECT or bv025-HECT as indicated. 48 h after transfection, cell lysates were subjected to immunoprecipitation (IP) using anti-FLAG antibody followed by WB analysis using antibody recognized RBM47. The cell lysates were also analyzed by WB using antibodies as indicated. nbRBM47-HECT inhibited the protein and mRNA expression of TSC22D3 in A549 cells. A549 cells were co-transfected with plasmids expressing UBE1L, UBCH8, and ISG15, plus FLAG-tagged nbRBM47-HECT or FLAG-bv025-HECT or FLAG-nbRBM47-HECT(C994A) as indicated for 48 h, followed by WB analysis (**C**) by using antibodies as indicated and RT-qPCR analysis of TSC22D3 mRNA expression normalized to Actb levels (**D**). **E** knockdown of RBM47 abolished the effect of nbRBM47-HECT on the induction of RBM47-ISGylation. siRNA RBM47 and scrambled siRNA were transfected into A549 cells as indicated. 48 h later, A549 cells were co-transfected with plasmids expressing UBE1L, UBCH8, and ISG15, plus FLAG-tagged nbRBM47-HECT or bv025-HECT as indicated for 24 h, followed by WB analysis by using antibodies as indicated. **F** nbRBM47-HECT inhibited TSC22D3 expression by inducing ISGylation of RBM47 in WT MEFs, but not in *R/R* MEFs. WT and *R/R* MEFs cells were co-transfected with plasmids expressing UBE1L, UBCH8, and ISG15, plus FLAG-tagged nbRBM47-HECT or bv025-HECT as indicated for 48 h, followed by WB analysis by using antibodies as indicated. **G** Jurkat T lymphocytes were co-transfected with plasmids expressing UBE1L, UBCH8, and ISG15, plus FLAG-tagged nbRBM47-HECT or bv025-HECT as indicated for 48 h, followed by WB analysis by using antibodies as indicated.

HERC5 is the only known human E3 ligase capable of catalyzing ISGylation by a catalytic HECT domain (homologous to E6-AP C terminus) with a cysteine 994 essential for its ligase activity [56]. To serve as a substrate binding domain, we fused nbRBM47 to the HECT domain (Fig. 5A). Strikingly, the resulting chimeric nbRBM47-HECT ligase increased RBM47-ISGylation in 293 T cells, whereas the control bv025-HECT failed to induce ISGylation (Fig. 5A). Co-immunoprecipitation assay showed that nbRBM47-HECT, but not bv025-HECT, induced the attachment of ISG15 to RBM47 (Fig. 5B). Consistently, nbRBM47-HECT increased ISGylation of RBM47 in A549 cells (Fig. 5C). Consequently, nbRBM47-HECT, but not bv025-HECT, inhibited the expression of TSC22D3 in A549 cells (Fig. 5C). By contrast, nbRBM47-HECT (C994A), a HECT active-site mutant deprived of potential ISG15 E3 ligase activity, did not affect RBM47-ISGylation or TSC22D3 expression (Fig. 5C). In addition, nbRBM47-HECT, but not bv025-HECT or nbRBM47-HECT (C994A), inhibited the mRNA expression of TSC22D3 in A549 cells (Fig. 5D). However, the knockdown of RBM47 abrogated the effect of nbRBM47-HECT on TSC22D3 expression in A549 cells (Fig. 5E). Consistently, nbRBM47-HECT augmented RBM47-ISGylation and subsequently decreased TSC22D3 expression in WT MEFs, but not in *R/R* MEFs lacking the ISGylation site (Fig. 5F). Furthermore, nbRBM47-HECT increased ISGylation of RBM47, along with decreased expression of TSC22D3 in Jurkat T cells, an immortalized human T lymphocyte cell line (Fig. 5G). Hence, our findings demonstrate that site-specific RBM47-ISGylation mediated by a chimeric E3 ligase diminishes TSC22D3 expression in various cell types, such as immune cells and human lung cancer cells.

### Epinephrine-mediated S309 phosphorylation primes K329 for ISGylation

**Fig. 6:**
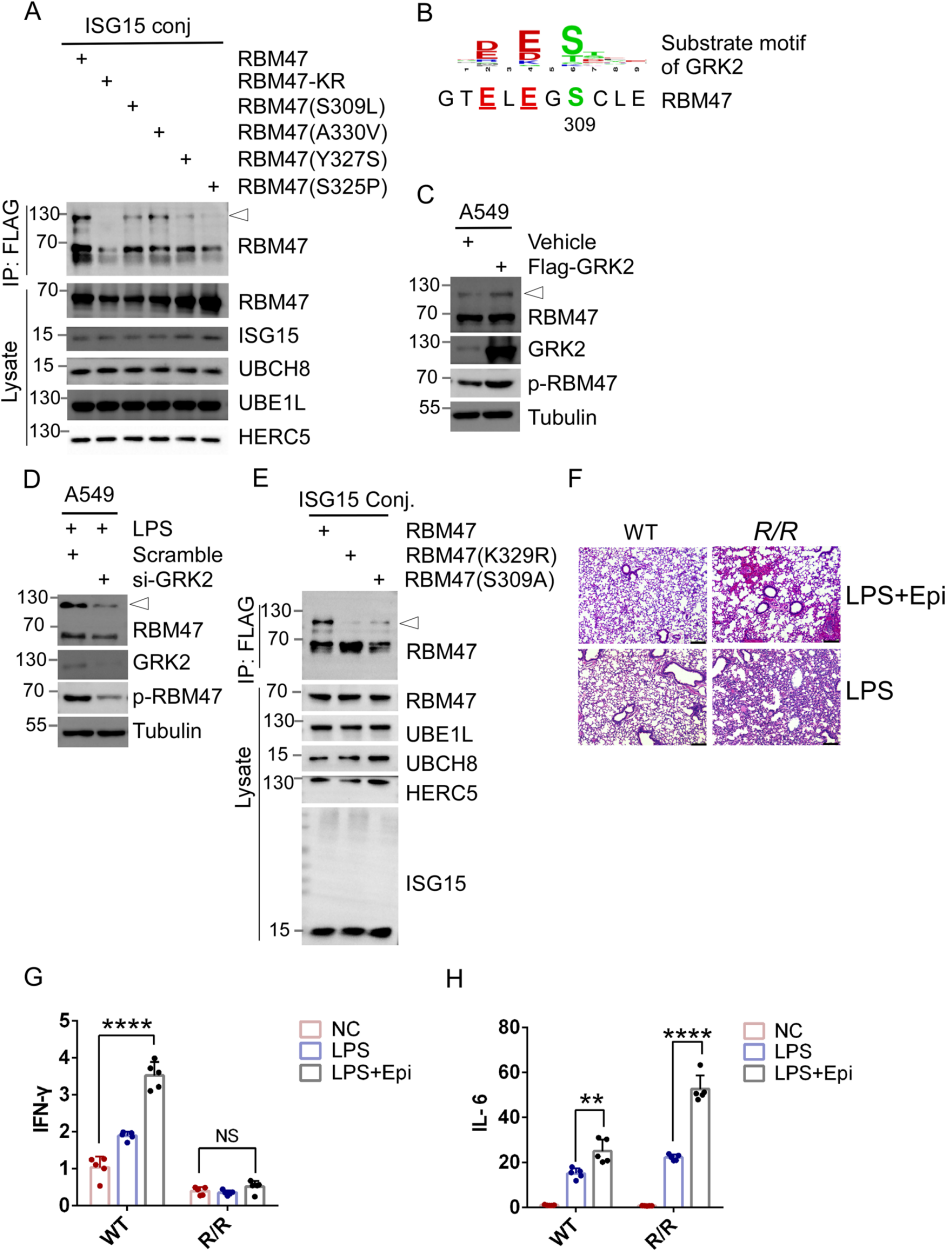
Epinephrine-mediated S309 phosphorylation primes K329 for ISGylation. **A** Human cancer-related mutations impaired RBM47-ISGylation. 293 T cells were co-transfected with plasmids expressing ISGylation modification enzymes, including UBE1L, UBCH8, HERC5, and FLAG-ISG15, plus plasmids expressing RBM47, RBM47-KR, S309L, A330V, Y327S, and S325P mutants. 48 h after transfection, cell lysates were subjected to IP using anti-FLAG magnetic beads followed by WB analysis using antibody recognized RBM47. And the cell lysates were analyzed by WB with indicated antibodies. **B** Serine 309 falls within a known consensus substrate motif of G protein-coupled receptor kinase 2 (GRK2): (D/E) X1-3(S/T) (where X represents any residue). **C** Overexpression of GRK2 promoted ISGylation of RBM47. A549 cells were mock-transfected and transfected with vector expressing GRK2 for 48 h, followed by WB analysis using antibodies as indicated. **D** Depletion of GRK2 inhibited ISGylation of RBM47. GRK2 siRNA and scrambled siRNA were transfected into A549 cells for 66 h, followed by LPS treatment (100 ng/ml) for 6 h, and WB analysis using antibodies recognized S309 phosphorylated RBM47 (*p*-RBM47), RBM47, GRK2, and Tubulin. **E** S309A impaired RBM47 ISGylation. 293 T cells were transfected with plasmids expressing ISGylation modification enzymes as in (**A**), plus plasmids expressing RBM47, K329R, and S309A mutants as indicated. 48 h after transfection, cell lysates were subjected to IP using anti-FLAG magnetic beads, followed by WB analysis using antibody recognized RBM47. The cell lysates were analyzed by WB with antibodies as indicated. **F** Epinephrine exaggerated LPS-induced ALI in *R/R* mice. LPS (2 mg/kg) were intraperitoneally injected into *R/R* and WT mice for 24 h, followed by intraperitoneal injection of epinephrine (2 mg/kg) for 30 min, then the lung tissues were collected for histological analysis with H&E staining (*n* = 5). Epinephrine increased LPS-induced mRNA expression of IFN-γ in WT mice but not in R/R mice. The experiments were done as in (**F**), and the mRNA expression levels of IFN-γ (**G**) and IL-6 (**H**) were examined by real-time quantitative reverse transcription PCR (RT-qPCR) on lung RNA isolated from R/R, R/WT, and WT mice. All samples were normalized using GAPDH housekeeping gene expression. Data were expressed as mean ± SEM. Statistical significance was determined by two-way mean ANOVA (*n* = 5 for each genotype; *, *p* value < 0.05, **, *p* value < 0.01, ***, *p* value < 0.001, ****, *p* < 0.0001).

We examined the Catalogue of Somatic Mutations in Cancer (COSMIC) database to investigate the possible association between RBM47-ISGylation and human disease. While we did not observe any mutations at the K329 locus, we identified several RBM47 mutations located adjacent to K329 in cancer patients, including S309L, S325P, Y327S, and A330V mutations [57]. To assess how these mutations affect RBM47-ISGylation, we overexpressed these RBM47 mutants, as well as FLAG-ISG15, UBE1L, UBCH8, and HERC5, in 293 T cells. ISGylated proteins were then isolated from the cell extracts by anti-FLAG immunoprecipitation (IP). IP results showed that the S309L, Y327S and S325P mutations reduced ISGylation of K329 to varying degrees (Fig. 6A). These results suggest that a linear motif around K329 affects RBM47-ISGylation.

We speculated that the serine 309 of RBM47 undergoes phosphorylation. To test this, we generated a phosphor-specific antibody capable of recognizing the phosphorylation consensus encompassing serine 309. WB results showed that this antibody (p-RBM47) recognized RBM47-S309D mutant with serine altered to the phosphorylation mimic aspartic acid, but not the S309A mutant with serine to unphosphorylable alanine mutation (Fig. S6A). RBM47 S309 phosphorylation levels were detectable in both A549 and 293 T cells, and alkaline phosphatase (CIP) treatment of PVDF membranes significantly reduced these levels. However, this dephosphorylation effect of CIP was reversed by the phosphatase inhibitors λ-PPase (Fig. S6B).

S309 resides within a substrate motif of GRK2 (G protein-coupled receptor kinase 2): (D/E) X1-3(S/T) (where X represents any residue) (Fig. 6B) [58]. GRK2, known as β-adrenergic receptor kinase, is activated by epinephrine. As expected, knockdown of GRK2 decreased S309 phosphorylation levels in A549 cells, whereas overexpression of GRK2 increased these levels (Fig. S6C, D). Overexpression of GRK2 in 293 T cells also increased S309 phosphorylation levels, while a dead GRK2 mutant (K220R) did not (Fig. S6E). Then, A549 cells were treated with the GRK2 agonist epinephrine (Epi) [59, 60] and the inhibitor paroxetine [61]. The results showed that epinephrine treatment increased S309 phosphorylation (Fig. S6F), whereas paroxetine treatment decreased S309 phosphorylation (Fig. S6G). Interestingly, overexpression of GRK2 increased the ISGylation of RBM47, along with an increased phosphorylation level of S309 (Fig. 6C). Conversely, knockdown of GRK2 inhibited LPS-induced RBM47-ISGylation, along with decreased phosphorylation level of S309 in A549 cells (Fig. 6D). Furthermore, anti-FLAG immunoprecipitation against FLAG-ISG15 revealed that the S309A mutation, like S309L, decreased the ISGylation of RBM47 (Fig. 6E). Our results suggest that the stress hormone epinephrine can induce ISGylation of RBM47 by activating its S309 phosphorylation.

Then, we investigated the impact of epinephrine on lung tissue in *K329R KI* mice. Treatment with low-dose epinephrine at 2 mg/kg for 30 min and 1 h elicited similar effects in *K329R KI* mice as observed in the wild-type (WT) mice, with a mild degree of congestion and exudation of lung tissue. However, acute administration of epinephrine (2 mg/kg for 30 min) considerably exacerbated LPS-induced acute lung injury in *R/R* mice compared to saline treatment, as demonstrated by collapsed alveolar morphology and pulmonary hemorrhage (Fig. 6F). Epinephrine is known to trigger cytokine release during the inflammatory process [62], as we observed in our study where acute epinephrine treatment potentiated the LPS-induced mRNA expression of *IFN-γ* and *IL-6* in WT mice. Intriguingly, the deficiency of RBM47-ISGylation in *R/R* mice under epinephrine stress resulted in a strikingly disordered expression of inflammatory factors upon LPS-treatment, with a further promotion of *IL-6* expression, with no difference observed in IFN-γ expression (Fig. 6G, H). These findings suggest a critical role of RBM47-ISGylation in modulating the inflammatory response under acute epinephrine stress.

## Discussion

ISGylation is widely known for its antiviral activity. However, its impact on tissue homeostasis and diseases like cancer remains poorly understood due to limited in vivo studies [7, 63–65]. Recently, the RNA-binding protein RBM47 was identified as a novel IFN-dependent ISG (IFN-stimulated genes) that has broad-spectrum antiviral activity by enhancing IFN-dependent immune response [38]. Our study further highlights RBM47 as an ISGylated protein, and defective ISGylation emerges as a key driver of multifaceted immunosuppression in the lung, promoting lung injury and tumorigenesis.

Interferons (IFNs) are a group of pleiotropic cytokines that regulate the production of chemokines or chemokine receptors to orchestrate tissue-immune homeostasis and promote antitumor immunity [42, 66]. Specifically, *CXCL9, CXCL10,* and *CCL5* have been identified as IFN-γ inducible chemokines [67, 68]. Our investigation in mice revealed that RBM47-ISGylation (K329R KI) deficiency led to reduced basal and LPS-induced *IFN-γ* mRNA expression, accompanied by a significant decrease in the expression of chemokines and chemokine receptors such as *CCR2*, *CCL5*, *CXCL9*, and *CXCL10*. These results suggest that RBM47-ISGylation plays a crucial role in regulating immune responses by modulating the expression of key chemokines and cytokines. Extracellular ISG15 has been shown to stimulate IFN-γ secretion by T and NK cells, while IFN-γ signaling components and the ISG15 gene are upregulated in breast cancer cells, implying a link between IFN-γ signaling and ISGylation [68, 69]. Our study provides the first evidence that ISGylation is essential for the basal and LPS-induced expression of IFN-γ, shedding light on the critical role of ISGylation in regulating immune responses and the importance of RBM47 in the modulation of IFN-γ expression. Additionally, IFN-γ signaling may play a critical role in cancer by mediating ISGylation [70]. Our study confirms their speculation by finding that IFN-γ induces ISGylation of RBM47 in vivo, thereby establishing a positive feedback loop between IFN-γ and RBM47-ISGylation that is likely to be critical for IFN responses in tissues.

Likewise, RBM47-ISGylation deficiency has been shown to suppress LPS-induced type I IFN expression, highlighting the multifaceted role of RBM47 ISGylation in regulating the immune response. Furthermore, deficiency of RBM47-ISGylation was found to increase the number of myeloid-derived suppressor cells (MDSCs) and decrease the abundance of tertiary lymphoid structures (TLSs) in lung tissues, both of which are associated with immunosuppression [43, 44, 50, 51]. Therefore, converging with previous studies on the implication of ISGylation or RBM47 in lung cancer [12, 23, 26, 71], it is plausible to propose that RBM47-ISGylation exerts tumor suppressor functions through its pleiotropic effects in activating antitumor immunity within the pulmonary microenvironment.

The deficiency of RBM47-ISGylation exacerbates LPS-induced acute lung injury (ALI) in *K329R KI* mice, with dysregulated cytokine expression observed, including elevated levels of pro-inflammatory cytokines such as *IL-6, CCL2*, and *GM-CSF*, and reduced levels of *IFN-γ*, *CCL5*, and *CXCL9*. *IL-6, CCL2*, and *GM-CSF*, as pro-inflammatory factors, have been upregulated as a marker in the pathogenesis of ALI [72]. Our findings reveal that RBM47-ISGylation regulates the mRNA expression of the immunosuppressive immune checkpoint, TSC22D3, as demonstrated by increased TSC22D3 expression in *K329R KI* mice, leading to inhibition of *IFN-γ* and related chemokines production. Conversely, knockdown of TSC22D3 restored the production of *IFN-γ* and related chemokines, leading to the amelioration of ALI in *K329R KI* mice. While TSC22D3 is commonly regarded as an anti-inflammatory therapeutic protein, reduced TSC22D3 expression has been reported to increase the release of pro-inflammatory factors and thus contribute to the development of inflammatory conditions such as colitis or liver fibrosis [33, 73]. However, the intricate role of TSC22D3 in inflamed tissue environments has been observed in studies wherein mice overexpressing TSC22D3 exhibited higher susceptibility to skin inflammation induced by imiquimod [74]. Similarly, aberrant induction of TSC22D3 has been observed to impair the suppressive function of Treg and contribute to enhanced intestinal inflammation [75]. Consistent to their observations, our study also suggests that immunosuppression upon TSC22D3 increase may exacerbate lung injury by impairing tissue repair or homeostasis. Furthermore, a deficiency of RBM47-ISGylation leads to TSC22D3-dependent immunosuppression while simultaneously increasing pro-inflammatory cytokines, thus cooperatively promoting ALI. RBM47 is an emerging regulator of immune responses by stabilizing the anti-inflammatory cytokine IL-10 [27]. Our study further suggests that RBM47-ISGylation may also confer a direct inhibitory effect on pro-inflammatory cytokine such as IL-6 expression, which is essential for maintaining a more balanced immune response. Nevertheless, further investigations are required to fully validate the mechanisms involved.

Recent studies have demonstrated that TSC22D3 can impair immune responses by inhibiting the type I interferon responses in dendritic cells and activation of *IFN-γ* + T cells, which hinders the anticancer immunosurveillance and immunotherapy of lung cancer [29]. Resonating with their observations, it is compelling that TSC22D3 plays a role in mediating lung carcinogenesis after RBM47-ISGylation deficiency via its inhibitory effect on the immune response. RBM47 is a novel molecular switch that determines cell fate by regulating the p53/p21-signaling axis [20]. Thus, further investigations of other effectors that may mediate the role of RBM47-ISGylation in preventing ALI or tumorigenesis in synergy with the immune response is warranted.

The lack of direct site-specific activation of ISGylation often hampers functional studies of ISGylation in cells or tissues. Our study offers a feasible solution by constructing chimeric E3 ligases by reprogramming the substrate recognition domain of the E3 ligase HERC5 using substrate-specific nanobodies. Our findings demonstrate that RBM47-ISGylation inhibits the expression of TSC22D3 in human cells, including lung cancer cell lines and the T lymphocyte line Jurkat, demonstrating that human RBM47-ISGylation has a consistent function, as confirmed by mouse models.

The interaction of ISGylation with other modifications or neighboring sequences remains unclear. Our findings discovered that RBM47 mutations in close proximity to K329, including S309L, S325P, Y327S, and A330V, observed in human cancers according to the COSMIC database, impair RBM47-ISGylation. This highlights the significance of ISGylation in human tissues. Remarkably, epinephrine-induced S309 phosphorylation by GRK2 kinase primes K329 for ISGylation. GRK2 is known to regulate β-adrenergic receptors that mediate the effects of (nor)epinephrine on various cell types [59, 60, 76]. Acute epinephrine treatment stimulates a stress response and trigger cytokine release during the inflammatory process [62, 77]. Although chronic stress is typically harmful due to its immunosuppressive effect, short-term or optimal stress, known as eustress, can be immunoenhancing and protect the organism through unknown mechanisms [78, 79]. Our findings demonstrate that epinephrine stress exacerbates the disordered expression of inflammatory factors in *R/R* mice, leading to increased exacerbation of lung injury. Hence, our study suggests that epinephrine-induced activation of RBM47-ISGylation may potentially enhance a protective immune response and maintain the equilibrium of inflammatory factor expression during acute stress.

In summary, our findings elucidate the potent immune activating function of RBM47-ISGylation that confers protective effects on lung tissue. Our findings also highlight the dynamic nature of ISGylation, which is influenced by epinephrine-induced phosphorylation. These findings offer crucial insights into the molecular mechanisms governing the immunological response in the lung, which could have significant implications for developing therapeutic interventions aimed at ameliorating lung diseases.

## Materials and methods

### Regents and plasmids

The phospho-specific antibody p-RBM47 was generated by immunizing rabbits with the synthetic phospho-peptide span serine 309 [mnnlngtelegs(p)clevtlakpvdkeq]. The RBM47 antibodies were generated by immunizing rabbits with the peptide [myggyagyipqafpaa]. Detailed information on the other antibodies, supplies, cloning strategies, and primers used in this study can be found in the supplemental materials.

### Cell lines and culture

HEK293T, A549, LEWIS lung carcinoma cells, and Jurkat immortalized T cell lines were obtained from American Type Culture Collection (ATCC) and China Center for Type Culture Collection. HEK293T, HCT116, CAL27, HepG2, and LEWIS lung carcinoma cells were cultured in DMEM supplemented with 10% FBS (hyclone), 100 units/ml penicillin, 100 µg/ml streptomycin. A549 cells and Jurkat T cell lines were maintained in RPMI with 10% FBS (hyclone), 100 units/ml penicillin, 100 µg/ml streptomycin. All cell lines were cultivated at 37 °C in 5% CO_2_ humidity. Early passage cell lines (<passage 25) were used. Plasmids or small interfering RNAs (siRNA) were transfected into cells according to Thermo Fisher protocol (L3000015, Lipofectamine 3000 Reagent). Efficient transfection of Jurkat T cell was performed by using Neon transfection system (Thermo Fisher Scientific) [80]. The siRNA sequences used in the study are listed in Supplementary Table S4.

### Mice

The RBM47 *K329R; K332R* knock-in mice were generated through CRISPR/Cas-mediated genome engineering on the C57BL/6 background by Cyagen Bio-sciences located in Guangzhou, China. All mice were housed in the animal facility at Huazhong Agricultural University. Mice were housed 4 per cage with food, and the housing environment was maintained at 22–24 °C with a 12 h light/dark schedule (lights on at 06:00 and off at 18:00). Mouse genotypes from tail biopsies were determined by PCR amplification with *SacII* enzyme digestion, for the *SacII* restriction site was present in the amplicon from the mutant allele. The primers were the forward primer 5′ GTACTCAACGTGATTGTATGC 3′ and the reverse primer 5′ CCTAAACTGGTAGGGCTTAATA 3′. The size of amplicons after *SacII* digestion is 702 bp for the wild-type allele and 465 bp, and 237 bp for the mutant allele. The mice were randomly allocated into groups before the experiment by utilizing computer software to generate random numbers, and different experimenters were responsible for mouse genotyping, mouse modeling experiments, and the evaluation of experimental results. All protocols for mice studies were approved by the Animal Experimentation Ethics Committee of Huazhong Agricultural University (Ethics No. HZAUMO-2021-0107 and HZAUMO-2022-0127).

### Western blotting analysis and co-immunoprecipitation (Co-IP)

Western blotting analysis was conducted following the standard protocol [81]. Cell lysates or tissue lysates suspended in 2 × SDS loading buffer were resolved by SDS-PAGE, transferred to a PVDF membrane, and probed with indicated antibodies. The immunoreactive bands were visualized by the enhanced chemiluminescence (Pierce) and quantified by densitometry with ChemiScope 6000 Exp (Chemi, CHN). The Immunoprecipitation assay was performed. Briefly, cells were lysed in 0.5% Triton lysis buffer (50 mM Tris pH 7.5, 150 mM NaCl, 0.5% Triton X-100, 2 mM EDTA) supplemented with the proteinase inhibitor cocktail (100 μg/ml), followed by incubation with 1 μg of antibody or control IgG. The immunocomplexes were brought down by protein A/G beads and subjected to Western blotting analysis.

### Urethane-induced lung adenocarcinoma model

Eight-week-old mice were administered an intraperitoneal injection of ethyl carbamate (urethane, U2500; Sigma) dissolved in 0.9% saline at a dose of 1 mg/g body weight per week for 10 weeks, with saline serving as the control. The mice were euthanized using isoflurane inhalation at either 12 or 8 weeks post-urethane treatment, and lung tissue specimens were collected. The left largest lobe of the lung tissue was utilized for HE staining to analyze histological features. The right upper lobe was employed for protein-related assays, while the right middle lobe was used for RNA-related assays.

### Statistical analysis

Statistical differences between two groups of variables were analyzed using two-tailed, paired, or unpaired Student’s *t* tests. For comparisons among more than two groups, one- or two-way analysis of variance (ANOVA) was performed, followed by Tukey’s multiple comparisons test to compare the means of three or more groups. SEM stands for Standard Error of the Mean. Statistical significance was considered at *p* values < 0.05. Lung tumor area quantification was carried out using ImageJ software. Statistical analysis was performed using Graphpad Prism 8.0 for data analysis and imaging.

### Supplementary information

original data

supplemental materials

## Data Availability

All data needed to evaluate the conclusions of this study are present in the manuscript. The RNA-Seq data: Gene Expression Omnibus (GEO) GSE218940. The MS data: PRIDE database, Project accession: PXD045984.
