# Supplementary material for: Dynamic RBM47 ISGylation confers broad immunoprotection against lung injury and tumorigenesis via TSC22D3 downregulation
Source: Cell Death Discov. 2023 Nov 30;9:430. doi: 10.1038/s41420-023-01736-z (PMC10689852; doi:10.1038/s41420-023-01736-z)
Supplement: Supplementary file 1 — original data [file 41420_2023_1736_MOESM1_ESM.pdf]

Fig1A

A549
+ Scramble
+ si-RBM47#1

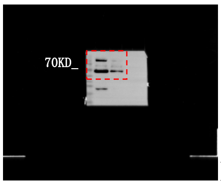

RBM47

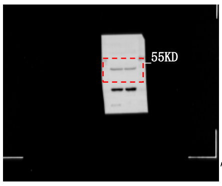

Tubulin

Fig1B

A549
+ Scramble
+ si-RBM47#2

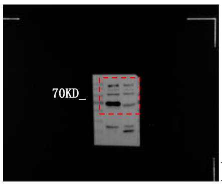

RBM47

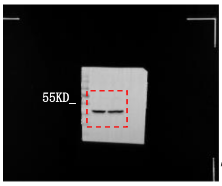

Tubulin

Fig1C

A549
+ Scramble
+ si-ISG15

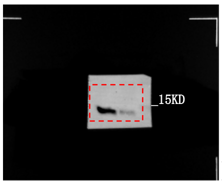

ISG15

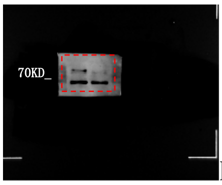

RBM47

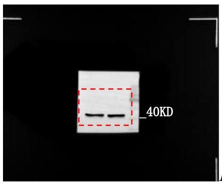

Actin

Fig1D

A549
+ Scramble
+ si-HERC5

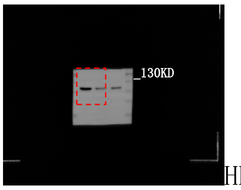

HERC5

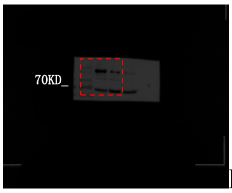

RBM47

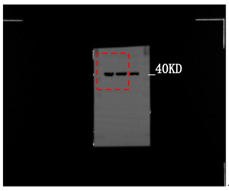

Actin

Fig1E

IP
Input
IgG
RBM47

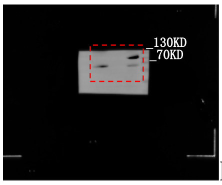

RBM47

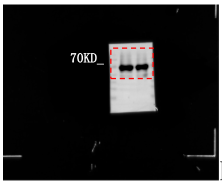

RBM47

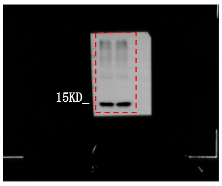

ISG15

Fig1F

ISG15 Conj.
+ RBM47
+ RBM47(G538A)
+ RBM47-CA
+ RBM47-KR

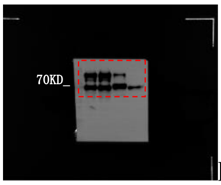

RBM47

Fig1G

ISG15 Conj.
+ RBM47
+ RBM47(K317R)
+ RBM47(K321R)
+ RBM47(K329R)
+ RBM47(K374R)

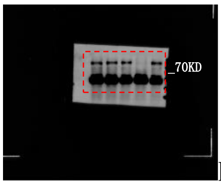

RBM47

Fig1H

ISG15 Conj.
+ RBM47
+ KR(R317K)
+ KR(R321K)
+ KR(R329K)
+ KR(R374K)

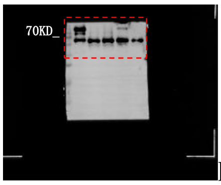

RBM47

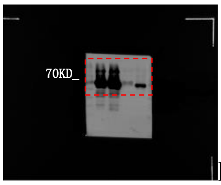 RBM47	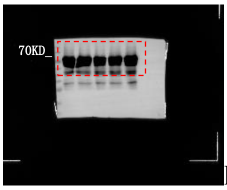 RBM47	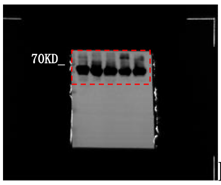 RBM47
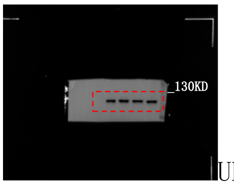 UBE1L	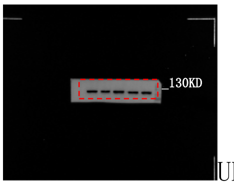 UBE1L	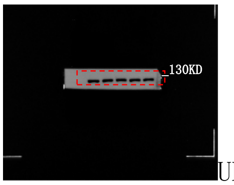 UBE1L
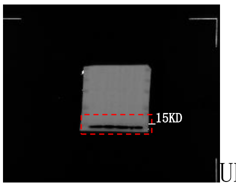 UBCH8	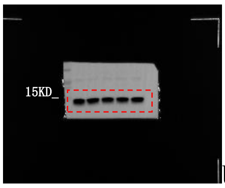 UBCH8	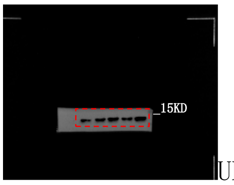 UBCH8
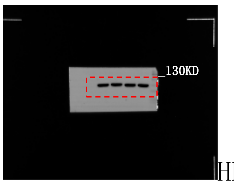 HERC5	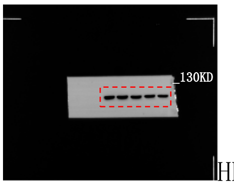 HERC5	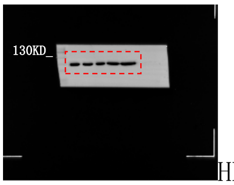 HERC5
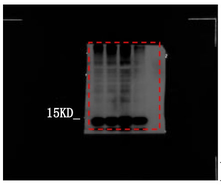 ISG15	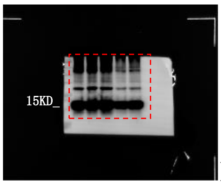 ISG15	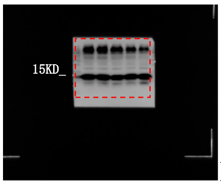 ISG15

Fig4A	Fig4B
A549<br>+ + Srcamble<br>+ si-RBM47#1<br>+ si-RBM47#2<br>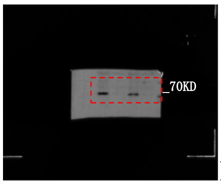 RBM47	A549 HEK293T<br>+ + Vehicle<br>+ + ISG conj<br>+ + + + RBM47<br>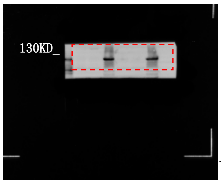 UBE1L
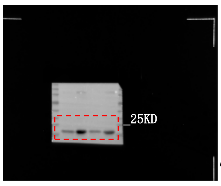 TSC22D3	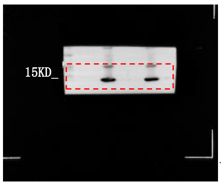 UBCH8

<table>
<tr>
<td rowspan="2">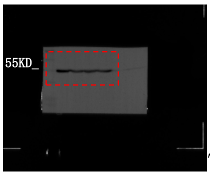

Tubulin</td>
<td>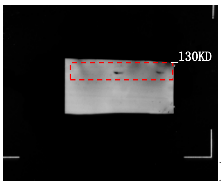

HERC5</td>
</tr>
<tr>
<td>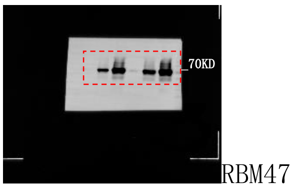

RBM47</td>
</tr>
<tr>
<td>Fig4C</td>
<td>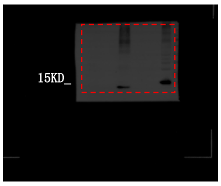

ISG15</td>
</tr>
<tr>
<td>Lung
WT R/WT R/R
+ + + LPS
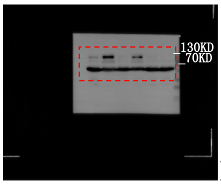

RBM47</td>
<td>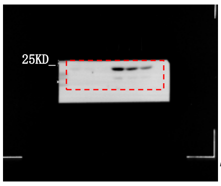

TSC22D3</td>
</tr>
<tr>
<td>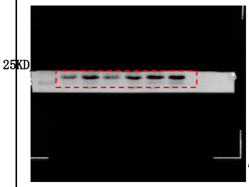

TSC22D3</td>
<td>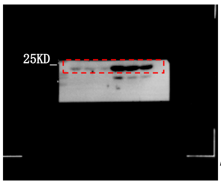

TSC22D3
(long exposure)</td>
</tr>
<tr>
<td>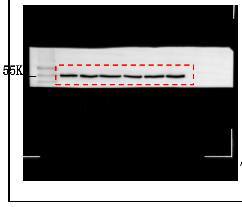

Tubulin</td>
<td>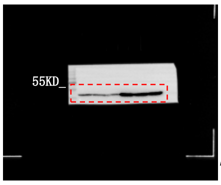

Tubulin</td>
</tr>
</table>

Fig5B	Fig5C
IP<br>+ HA-bv025-HECT<br>+ HA-nbRBM47-HECT<br>+ + UBE1L, UBCH8, FLAG-ISG15<br>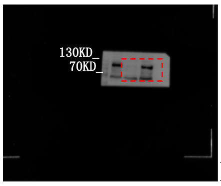<br>RBM47	A549<br>+ bv025-HECT<br>+ nbRBM47-HECT<br>+ nbRBM47-HECT(C994A)<br>+ + + UBE1L, UBCH8, ISG15<br>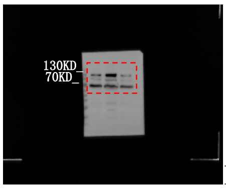<br>RBM47
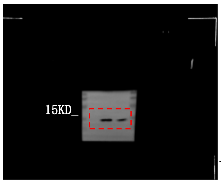<br>UBCH8	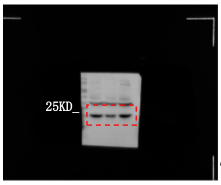<br>TSC22D3
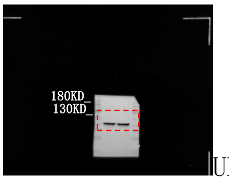<br>UBE1L	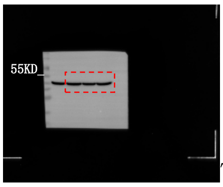<br>Tubulin
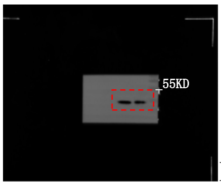<br>HA	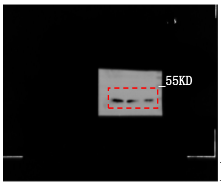<br>Flag
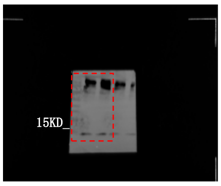<br>ISG15	

Fig5E	Fig5F	Fig5G
A549<br>+ + Scramble<br>+ + si-RBM47<br>+ + bv025-HECT<br>+ + nbRBM47-HECT<br>+ + + + UBE1L, UBCH8, ISG15<br>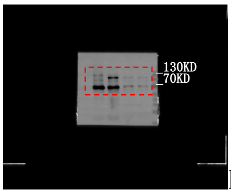<br>RBM47	MEF<br>WT *R/R*<br>+ + bv025-HECT<br>+ + nbRBM47-HECT<br>+ + + + UBE1L, UBCH8, ISG15<br>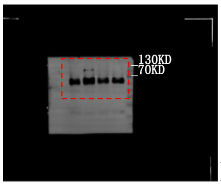<br>RBM47	Jurkat T<br>+ bv025-HECT<br>+ nbRBM47-HECT<br>+ + UBE1L, UBCH8, ISG15<br>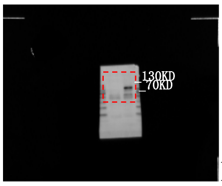<br>RBM47
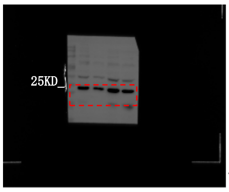<br>TSC22D3	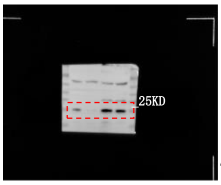<br>TSC22D3	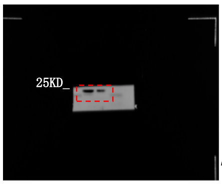<br>TSC22D3
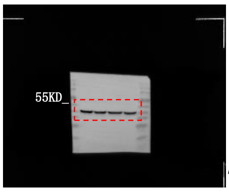<br>Tubulin	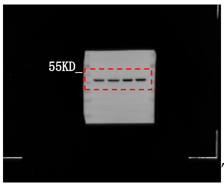<br>Tubulin	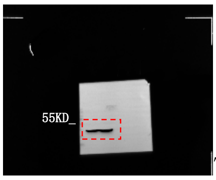<br>Tubulin
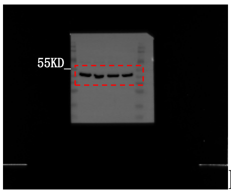<br>Flag	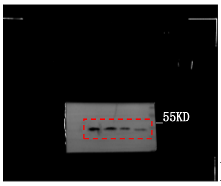<br>Flag	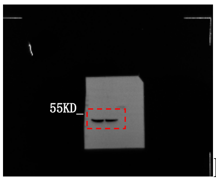<br>Flag

Fig6A	Fig6E
ISG15 conj<br>+ RBM47<br>+ RBM47-KR<br>+ RBM47(S309L)<br>+ RBM47(A330V)<br>+ RBM47(Y327S)<br>+ RBM47(S325P)<br>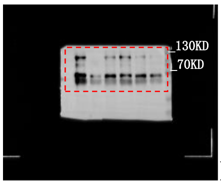<br>RBM47	ISG15 Conj.<br>+ RBM47<br>+ RBM47(K329R)<br>+ RBM47(S309A)<br>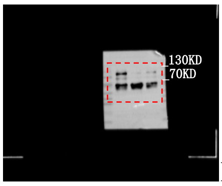<br>RBM47
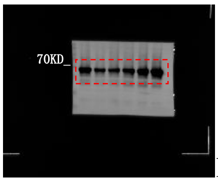<br>RBM47	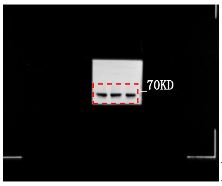<br>RBM47

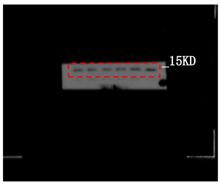<br><br>ISG15	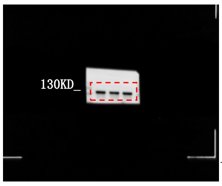<br><br>UBE1L
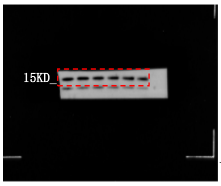<br><br>UBCH8	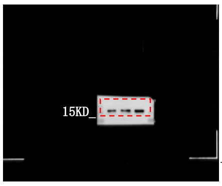<br><br>UBCH8
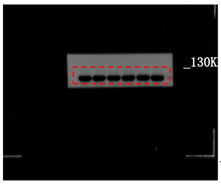<br><br>UBE1L	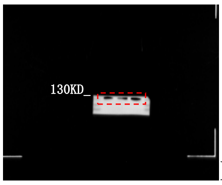<br><br>HERC5
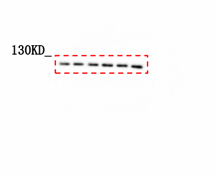<br><br>HERC5	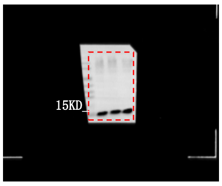<br><br>ISG15

Fig6C	Fig6D
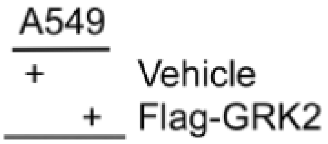<br><br>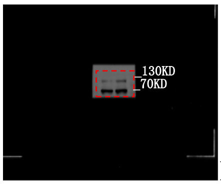<br><br>RBM47	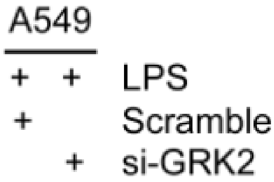<br><br>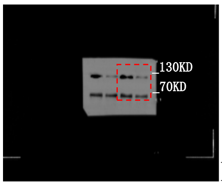<br><br>RBM47
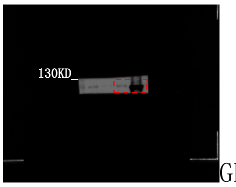<br><br>GRK2	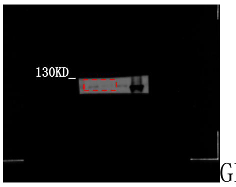<br><br>GRK2
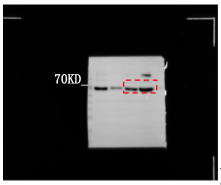<br><br>p-RBM47	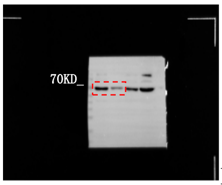<br><br>p-RBM47

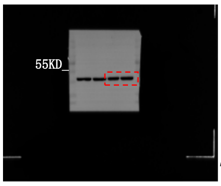 Tubulin	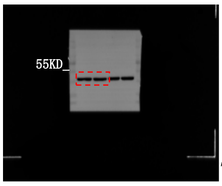 Tubulin

SFig1A	SFig1C	SFig1D
Cell Line<br>1 2 3 4<br>1-A549<br>2-HCT116<br>3-CAL27<br>4-HepG2<br>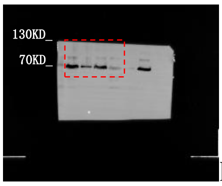 RBM47	A549<br>0 3 6 LPS (h)<br>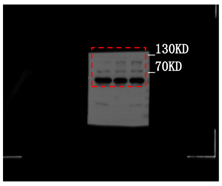 RBM47	A549<br>0 50 100 LPS (ng/ml)<br>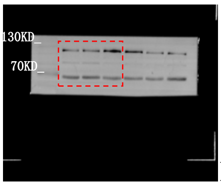 RBM47
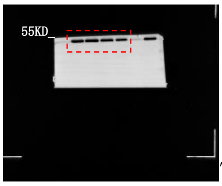 Tubulin	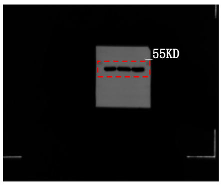 Tubulin	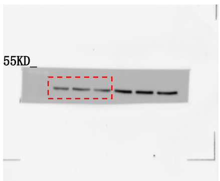 Tubulin

SFig1E	SFig1F	SFig1G
A549<br>+ Scramble<br>+ si-UBE1L<br>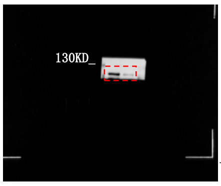 UBE1L	A549<br>+ Scramble<br>+ si-UBCH8<br>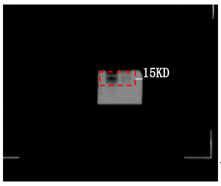 UBCH8	ISG15 Conj.<br>+ + RBM47<br>+ USP18<br>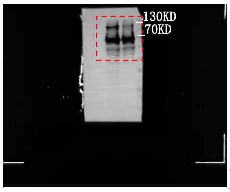 RBM47
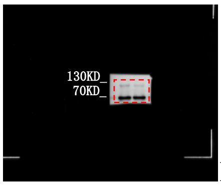 RBM47	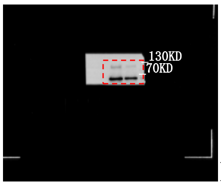 RBM47	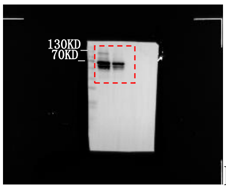 RBM47

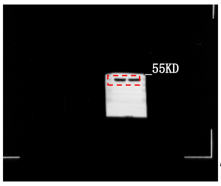

Tubulin

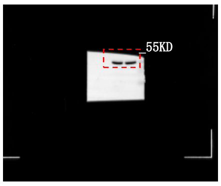

Tubulin

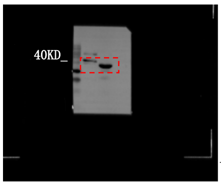

USP18

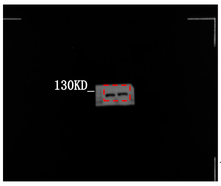

UBE1L

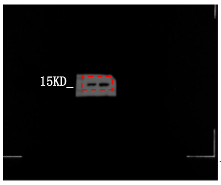

UBCH8

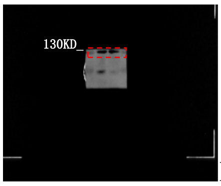

HERC5

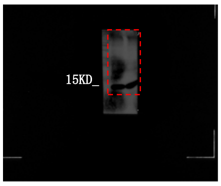

ISG15

SFig2B

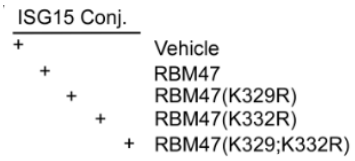

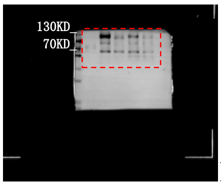

RBM47

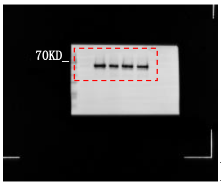

RBM47

SFig2D

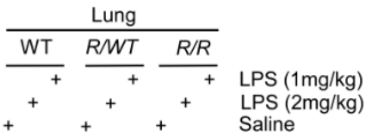

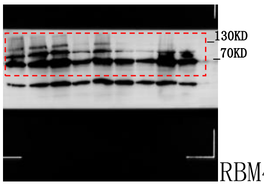

RBM47

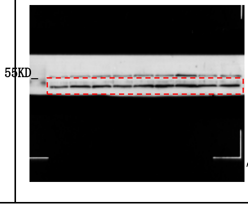

Tubulin

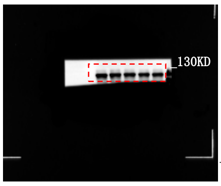 UBE1L	
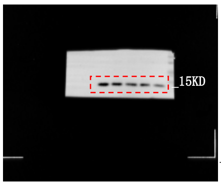 UBCH8	
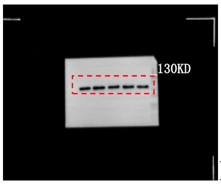 HERC5	
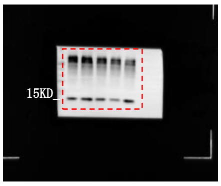 ISG15	
SFig2M	SFig2N
lung<br>0 0.3 1 3 IFN-γ (μg/kg)<br>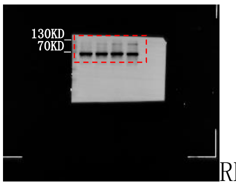 RBM47	A549<br>+ + LPS<br>+ Scramble<br>+ si-IFN-γ<br>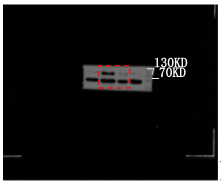 RBM47
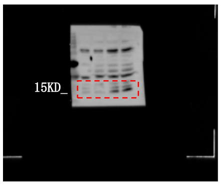 IFN-γ	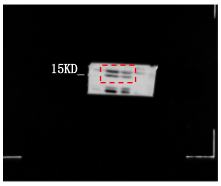 IFN-γ
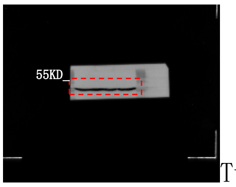 Tubulin	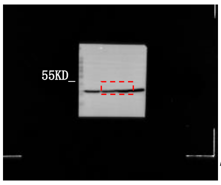 Tubulin

SFig4D	SFig4F	SFig4G
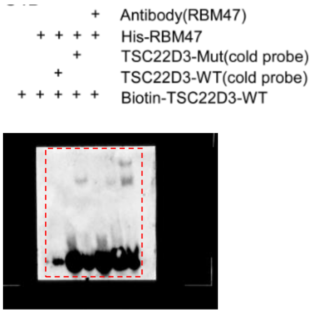	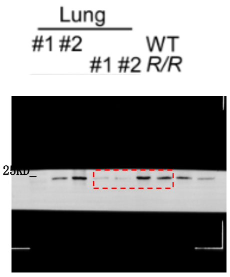<br>TSC22D3	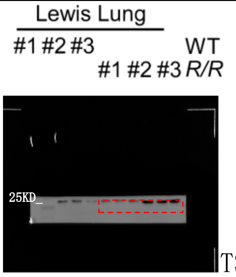<br>TSC22D3
	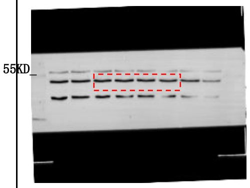<br>Tubulin	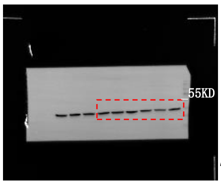<br>Tubulin

SFig4H	SFig4I
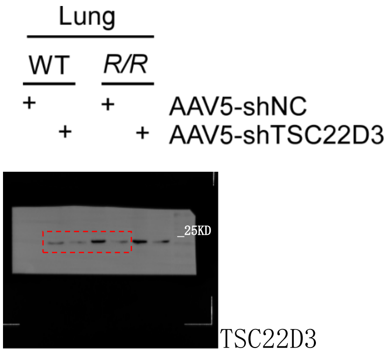<br>TSC22D3	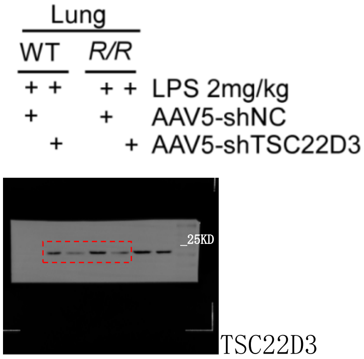<br>TSC22D3
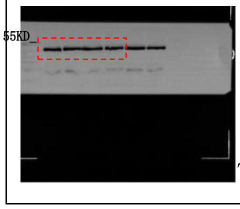<br>Tubulin	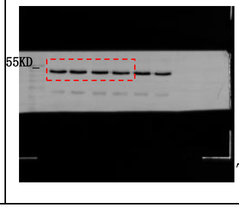<br>Tubulin

SFig5B	SFig5C
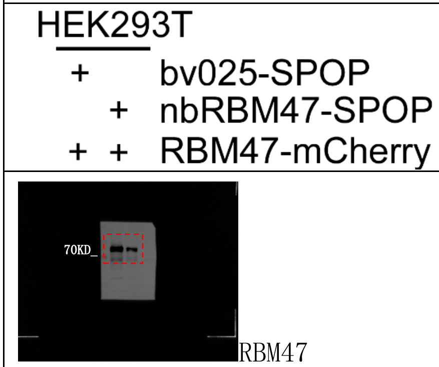<br>RBM47	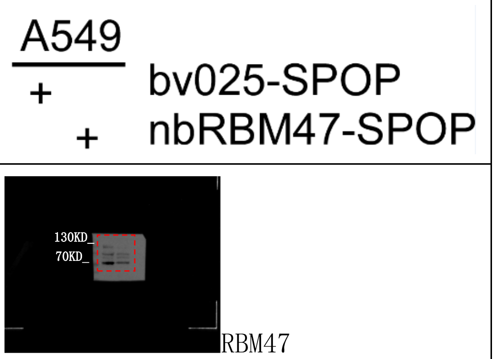<br>RBM47

<table>
<tr><td>
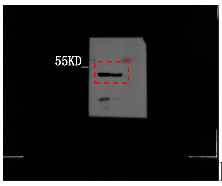

Flag
</td><td>
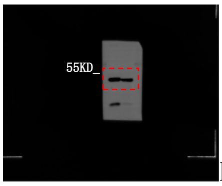

Flag
</td></tr>
<tr><td>
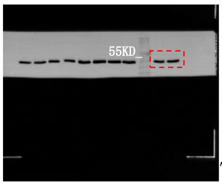

Tubulin
</td><td>
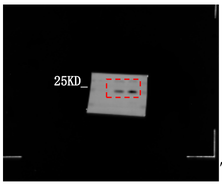

TSC22D3
</td></tr>
<tr><td></td><td>
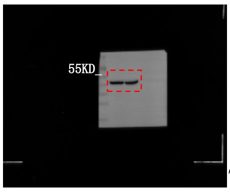

Tubulin
</td></tr>
</table>

<table>
<tr><td>SFig6A</td><td>SFig6B</td></tr>
<tr><td>
HEK293T
+ Vehicle
+ RBM47
+ RBM47S309A
+ RBM47S309D
</td><td>
A549 HEK293T
+ + + + CIP
+ + PIC
</td></tr>
<tr><td>
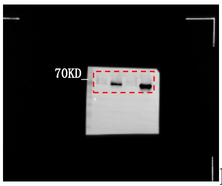

p-RBM47
</td><td rowspan="2">
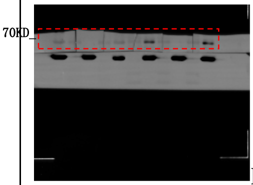

p-RBM47
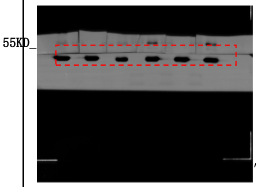

Tubulin
</td></tr>
<tr><td>
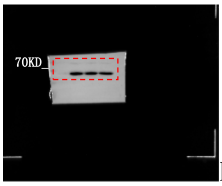

RBM47
</td></tr>
<tr><td>
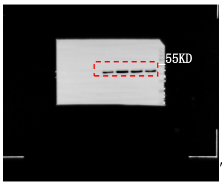

Tubulin
</td><td></td></tr>
</table>

SFig6C	SFig6D
A549<br>+ Scramble<br>+ si-GRK2	A549<br>+ Vehicle<br>+ Flag-GRK2
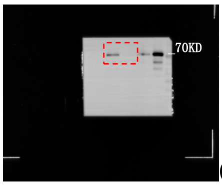 GRK2	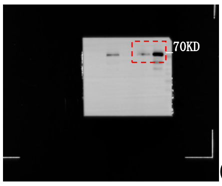 GRK2
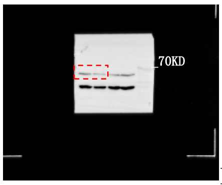 p-RBM47	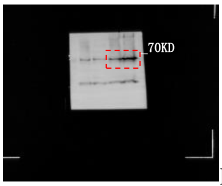 p-RBM47
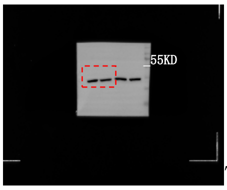 Tubulin	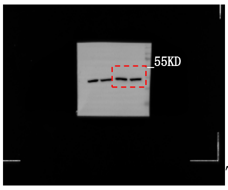 Tubulin

SFig6E	SFig6F	SFig6G
HEK293T<br>+ Vehicle<br>+ Flag-GRK2<br>+ Flag-GRK2(K220R)	A549<br>0 0.5 1 Epi (h)	A549<br>0 0.5 1 Paroxetine(h)
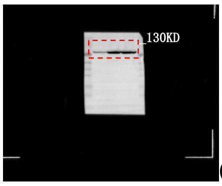 GRK2	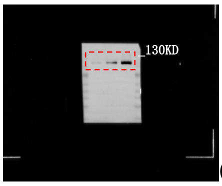 GRK2	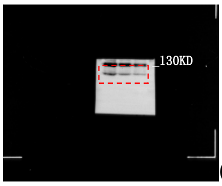 GRK2
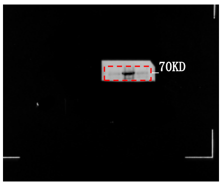 p-RBM47	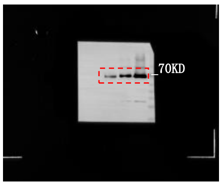 p-RBM47	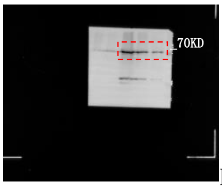 p-RBM47
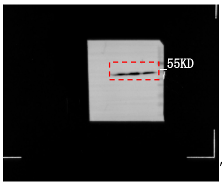 Tubulin	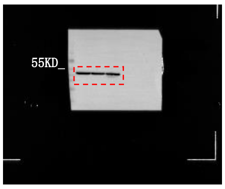 Tubulin	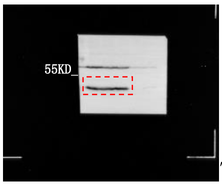 Tubulin
