# Supplementary material for: Dynamic RBM47 ISGylation confers broad immunoprotection against lung injury and tumorigenesis via TSC22D3 downregulation
Source: Cell Death Discov. 2023 Nov 30;9:430. doi: 10.1038/s41420-023-01736-z (PMC10689852; doi:10.1038/s41420-023-01736-z)

Supplementary Information for

Dynamic RBM47 ISGylation confers broad immunoprotection against lung injury and tumorigenesis via TSC22D3 downregulation

Materials and Methods

Reagent or Resource

The used reagent and resource were listed in Table S1.

Plasmids

The used PCR-Primers were listed in Table S2.

Histopathology

Lung tissue samples were fixed overnight with 4% paraformaldehyde at room temperature. Paraffin sections were prepared from the fixed tissue and stained with hematoxylin and eosin (H&E) to determine morphology and inflammation. Immunohistochemistry was performed on paraffin sections of the lungs using the TSC22D3 antibody (ABclonal A19911).For histology scoring, an acute lung injury (ALI) scoring system was developed with reference to the criteria of Osman[1] and Hofbauer[2] (Table S3).

Immunoprecipitation (IP) with anti-FLAG M2 magnetic beads

The methodology employed for immunoprecipitation was performed in accordance with previously established procedures[3]. Briefly, 293T cells were seeded to achieve confluence ranging from 60% to 70% in 6-well plates, after which a total of 5 μg of the plasmid was transfected. After 24 hours of transfection, cells were washed once with PBS and subsequently lysed using a buffer solution containing 25 mM Tris-HCl pH 7.5, 150 mM NaCl, 10% glycerol, and 1% TritonX-100, supplemented with a protease inhibitor mixture (Roche Molecular Biochemicals). Precleared lysates were subjected to anti-FLAG M2 magnetic beads as per the manufacturer's guidelines (F1804 sigma). The beads were washed four times with the aforementioned lysis buffer, and the immunoprecipitates were eluted using SDS sample buffer, followed by immunoblot analysis.

Mass Spectrometry (LC-MS/MS)

Purification of the target proteins was accomplished by FLAG-immunoprecipitation (FLAG-IP) from cells expressing RBM47. Subsequently, the purified proteins were separated using 10% SDS-PAGE gel, and subjected to Coomassie brilliant blue R-250 (BioFroxx) staining at room temperature for 1 hour to facilitate the determination of protein distribution on the lanes. After decolorizing, the full-size lanes were excised and transferred to Gene Create Bioengineering Co. Ltd. (Wuhan, China), where the samples were processed in accordance with standard procedures. The analytical evaluation was carried out using Triple TOF™ 5600 plus (AB Sciex Pte. Ltd, Singapore). Mascot Daemon was utilized for database search analysis and the MS data was acquired.

bioPROTACs assay

The engineered fusion protein bioPROTAC (bioproteolytic targeting chimera) was expressed in cells to drive targeted degradation of the target protein[4]. The E3 ligase was subjected to modification through the direct replacement of its substrate recognition domain with Nanobody (nb.RBM47 or nb.bv025), which specifically binds to the target protein. 293T cells were cultured in 12-well plates and subsequently transfected with plasmid pcDNA3-RBM47-mcherry. Following a 24-hour incubation period, bioPROTACs plasmids were transfected into HEK-293T cells, and the cells were allowed to incubate for a further 48 hours. The efficiency of the process was subsequently evaluated via immunoblot analysis.

RNA-Seq

Lung tissue samples were obtained from 8-week-old WT and R/R littermate mice and used for RNA-sequencing. The total RNA was extracted utilizing Trizol reagent as per the manufacturer's instructions (Thermo Fisher Scientific). Each sample group comprised three biological replicates.

According to the manufacturer's protocol (Illumina), stranded paired-end RNA sequencing (RNA-seq) with 150 bp read lengths was performed using a HiSeq2500. The acquired sequence reads were aligned to the mouse genome sequence (mm10) by HISAT2 (version 2.1.0)[5]. The number of reads per gene was determined using feature Counts v1.6.2 (WT vs. *R/R* samples)[6]. Differentially expressed genes were determined using edgeR[7]. GSEA analysis were performed using the cluster-profiler[8]. MCODE analysis for protein-protein interaction enrichment was performed using the Metascape online tool (www.metascape.org)[9].

LPS-induced lung injury

Intraperitoneal injections were administered to mice utilizing lipopolysaccharide (LPS) (from E. coli O111:B6 sigma) dissolved in saline, with doses of 1 mg/kg or 2 mg/kg, and the same volume of saline was utilized as a control. At the end of 24 hours after the intraperitoneal injection, all mice were euthanized via inhalation of isoflurane, following which their lungs were extracted for further experimentation.

Mice were subjected to nebulized LPS or vehicle only (control group), as previously described[10]. Briefly, the animals were exposed to nebulized LPS or saline for the vehicle control, and lung tissue was collected 24 hours (LPS 24h) after the nebulized LPS-induced lung injury. The nebulization protocol involved the administration of a total volume of 5 mL of LPS solution at a concentration of 3 mg/mL (Lipopolysaccharides from Escherichia coli 0111:B4 – Sigma-Aldrich), with sufficient time for complete solution nebulization (mean of 30 minutes). The control group received 5 mL of saline solution containing 0.9% NaCl.

Mouse model of Lewis lung carcinoma

The mouse Lewis lung cancer cell line (LLC) was intravenously administered to C57BL/6 mice at a dose of 0.25×10^6 cells per mouse via the tail vein. After three weeks of the injection, the mice were humanely euthanized by inhalation of isoflurane and the lungs were carefully extracted for further experiments.

RT-qPCR analysis

Reverse transcription was conducted using 1 μg of total RNA and ABScript II cDNA First-Strand Synthesis Kit (ABclonal) as per the manufacturer's instructions. The primer sets employed in the study have been listed in Supplementary Table S5. Real-time quantitative reverse transcriptase PCR (RT-qPCR) was utilized to measure the mRNA expression of cytokines. The RT-qPCR was conducted following the Vazyme, ChamQ Universal SYBR® qPCR Master MIX protocol (vazyme).

Production of adeno-associated viruses

The production of AAV5-shTSC22D3 and control AAV5-shNC strains was performed by Cyagen (Suzhou, China) and involved screening of DNA sequencing. The AAV plasmid was subsequently packaged into AAV serotype 5 virus by Cyagen (Suzhou, China). Following production, AAV5-shTSC22D3 (8.21×10^{12} GC/ml) or control AAV5-shNC (7.84×10^{12} GC/ml) were administered to the lungs via tracheal intubation and mice were treated for 6 weeks. The viral titer is expressed as Genomic Copies per mL (GC/ml).

RNA-IP

To investigate RNA-protein interactions, RNA-immunoprecipitations (RNA-IP) were conducted according to previously published protocols[11]. Cell extracts were prepared using an immunoprecipitation buffer comprising of 100 mM KCl, 5 mM MgCl2, 10 mM HEPES, 1 mM 1,4-Dithiothreitol (DTT), and 0.5% NP-40. The extracts were then incubated with 1 μg of anti-RBM47 or an isotype control IgG overnight at 4°C. The RNA–protein immunocomplexes were isolated by protein A/G beads and subjected to RT-PCR analysis. The semiquantification of primers used for RT-PCR analysis were elaborated in supplemental Table S5.

RNA Electrophoretic Mobility Shift Assay (RNA EMSA)

TSC22D3-WT (5′-AAAUGCGUAUAUAUACCCAU-3′) and TSC22D3-Mut (5′-AAGCGCACAAGCCACGCCAC-3′) RNA sequences were synthesized by using a mMessage mMachine SP6 in vitro transcription kit (Invitrigen). Biotin 3' end RNA labeling and purification (5′-AAAUGCGUAUAUAUACCCAU-3′-Biotin) were performed according to Pierce™ RNA 3' end Biotinylation Kit instructions (Thermo, 20160). His-RBM47 proteins were incubated with Biotin labeled TSC22D3-WT RNA in the presence or absence of cold TSC22D3-WT and cold TSC22D3-Mut, as indicated for each experiment. A gel shift assay was conducted using a previously reported protocol[12].

The construction of a synthetic yeast display nanobody library

The construction of the nanobody library followed the previously described protocol.[13] The DNA library pool of nanobodies was amplified for yeast transformations, and a yeast library was subsequently generated with a capacity of 2.5×10^{9}.

The peptide utilized for RBM47 nanobody screening was a K329 spanning peptide with the sequence CKEQYSRYQKAA. The Bovine Serum Albumin (BSA) fused peptide was diluted in FACS buffer (PBS, pH 7.4, 10 mM EDTA) and preincubated with anti-BSA antibody (mouse, 1:200) for 1 hour at room temperature. After galactose induction of nanobodies, 1×10^{10} yeast cells were resuspended in FACS buffer and incubated with pretreated BSA-peptide for 1 hour at room temperature, followed by washing with FACS buffer. Yeast displaying nanobodies were incubated with anti-myc antibody, labeled with anti-rabbit IgG-Alexa Fluor 647 (1:200), and pretreated BSA-peptide was labeled with anti-mouse IgG-FITC (1:50). Yeast populations carrying dual fluorescence of FITC and Alexa Fluor 647 were enriched by flow cytometry with unlabeled yeast cells as negative control. Four rounds of flow cytometry screening were performed with sequential ten-fold decreases in antigen (BSA-peptide) concentration from 10μM to 10pM.

The CDR (complementarity-determining region) sequence of nanobody nbRBM47 is ……GRTFSSYA(CDR1)……SRGGSRTN(CDR2)……RYGDYPSSSASYYNY (CDR3)……

Flow Cytometry

Single-cell suspensions of lungs were prepared according to previously described methods.[14] After euthanizing the mice, lung tissues were rinsed with PBS, digested with 1.5 mg/mL Collagenase A and 0.4 mg/mL DNaseI, and then filtered to create a single-cell suspension. Red blood cell fragments were treated with a lysis solution. After cell counting, $1\text{x}10^{6}$ cells per sample were resuspended with 100ul FC flow buffer (Thermo Fisher). CD16/CD32 antibody (eBioscience) was added and incubated at 4°C for 10 min. After washing, cells were stained with a panel of standard immunophenotyping antibodies for 30 min at room temperature. Following staining, cells were washed and fixed with 0.4% paraformaldehyde in PBS. Data were acquired using a Cytoflex-LX flow cytometer and compensation was performed at the beginning of each experiment. Data were analyzed using FlowJo v10 software.

References

1 Osman M. O., Kristensen J. U., Jacobsen N. O., Lausten S. B., Deleuran B., Deleuran M. et al. A monoclonal anti-interleukin 8 antibody (WS-4) inhibits cytokine response and acute lung injury in experimental severe acute necrotising pancreatitis in rabbits. Gut. 1998; 43: 232-239.

2 Hofbauer B., Saluja A. K., Bhatia M., Frossard J. L., Lee H. S., Bhagat L. et al. Effect of recombinant platelet-activating factor acetylhydrolase on two models of experimental acute pancreatitis. Gastroenterology. 1998; 115: 1238-1247.

3 Zou W.,Zhang D. E. The interferon-inducible ubiquitin-protein isopeptide ligase (E3) EFP also functions as an ISG15 E3 ligase. J Biol Chem. 2006; 281: 3989-3994.

4 Lim S., Khoo R., Peh K. M., Teo J., Chang S. C., Ng S. et al. bioPROTACs as versatile modulators of intracellular therapeutic targets including proliferating cell nuclear antigen (PCNA). Proc Natl Acad Sci U S A. 2020; 117: 5791-5800.

5 Kim D., Langmead B.,Salzberg S. L. HISAT: a fast spliced aligner with low memory requirements. Nat Methods. 2015; 12: 357-360.

6 Liao Y., Smyth G. K.,Shi W. featureCounts: an efficient general purpose program for assigning sequence reads to genomic features. Bioinformatics. 2014; 30: 923-930.

7 Robinson M. D., McCarthy D. J.,Smyth G. K. edgeR: a Bioconductor package for differential expression analysis of digital gene expression data. Bioinformatics. 2010; 26: 139-140.

8 Yu G., Wang L. G., Han Y.,He Q. Y. clusterProfiler: an R package for comparing biological themes among gene clusters. Omics. 2012; 16: 284-287.

9 Zhou Y., Zhou B., Pache L., Chang M., Khodabakhshi A. H., Tanaseichuk O. et al. Metascape provides a biologist-oriented resource for the analysis of systems-level datasets. Nat Commun. 2019; 10: 1523.

10 de Souza Xavier Costa N., Ribeiro Júnior G., Dos Santos Alemany A. A., Belotti L., Zati D. H., Frota Cavalcante M. et al. Early and late pulmonary effects of nebulized LPS in mice: An acute lung injury model. PLoS One. 2017; 12: e0185474.

11 Peritz T., Zeng F., Kannanayakal T. J., Kilk K., Eiríksdóttir E., Langel U. et al. Immunoprecipitation of mRNA-protein complexes. Nat Protoc. 2006; 1: 577-580.

12 Rio D. C. Electrophoretic mobility shift assays for RNA-protein complexes. Cold Spring Harb Protoc. 2014; 2014: 435-440.

13 McMahon C., Baier A. S., Pascolutti R., Wegrecki M., Zheng S., Ong J. X. et al. Yeast surface display platform for rapid discovery of conformationally selective nanobodies. Nat Struct Mol Biol. 2018; 25: 289-296.

14 Yu Y. R., O'Koren E. G., Hotten D. F., Kan M. J., Kopin D., Nelson E. R. et al. A Protocol for the Comprehensive Flow Cytometric Analysis of Immune Cells in Normal and Inflamed Murine Non-Lymphoid Tissues. PLoS One. 2016; 11: e0150606.

Supplementary Tables

Table S1 Reagent or Resource

REAGENT	SOURCE	IDENTIFIER
**Antibodies**		
β-tubulin	Sigma Aldrich	T5201
HERC5	ABclonal	A14889
GRK2	ABclonal	A4443
HA	ABclonal	AE008
TSC22D3	ABclonal	A19911
UBA7	ABclonal	A9142
FLAG	Sigma Aldrich	F4799
ISG15	ABclonal	A1182
IFN-γ	ABclonal	A12450
UBE2L6	ABclonal	A13670
HRPAnti-RabbitIgGLight Chain	ABclonal	AS061
HRPAnti-MouseIgGLight Chain	ABclonal	AS062
Mouse Control IgG	ABclonal	AC011
Rabbit Control IgG	ABclonal	AC005
HRP Goat Anti-Rabbit IgG	ABclonal	AS014
HRP Goat Anti-Mouse IgG	Abclonal	AS003
ly6g-APC	Thermo Fisher	17-9668-80
cd11b-PE-CY7	Thermo Fisher	25-0118-41
cd45	Thermo Fisher	48-0451-82
cd11b	Thermo Fisher	25-0118-41
cd38	BioLegend	102707
f4/80	BioLegend	123129
cd4	BioLegend	100425
**Kit**		
Bradford Protein Assay	Beyotime	P0006C
Tissue Protein Extraction	CWBIO	CW0891M
**Chemicals**		
Epinephrine	MedChemExpress	HY-B0447A
Paroxetine hydrochloride	MedChemExpress	HY-B0492
Urethane	Sigma	U2500
LPS	Sigma	L2630

Table S2 PCR-Primers for Genes

Gene ID No	Forward primer (5'–3')	Reverse primer (5'–3')
**GRK2**	AAAAGGATCCATGGCGGACCTGGAGG	AAAACTCGAGTCAGAGGCCGTTGGCAC
**RBM47**	AAAAGGATCCATGACCGCAGAGGATTCCAC	AAAACTCGAGTCAGTATGTCTGGTAGACGT
**RBM47-KR**	AAAAGGATCCATGACCGCAGAGGATTCCAC	AAAACTCGAGTCAGTATGTCTGGTAGACGT
**RBM47 -CA**	AAAAGGATCCATGACCGCAGAGGATTCCAC	AAAACTCGAGTCAGTATGTCTGGTAGACGT
**RBM47-SA mutant (S309A)**	AAAAAAGCTTATGACCGCAGAGGATTCCAC	AAAACTCGAGTCAGTATGTCTGGTAGACGT
	GGCACTGAGCTGGAGGGCGCTTGCCTGGAGGTCACGCTG	CAGCGTGACCTCCAGGCAAGCGCCCTCCAGCTCAGTGCC
**RBM47-SD mutant (S309D)**	AAAAAAGCTTATGACCGCAGAGGATTCCAC	AAAACTCGAGTCAGTATGTCTGGTAGACGT
	GGCACTGAGCTGGAGGGCGATTGCCTGGAGGTCACGCTG	CAGCGTGACCTCCAGGCAATCGCCCTCCAGCTCAGTGCC
**RBM47(G538A)**	AAAAAAGCTTATGACCGCAGAGGATTCCACCGC	AAAACTCGAGTCAGTATGTCTGGTAGACGTCGGGG
	GCCGGGATCTACCGGGCCAGTTA	TAACTGGCCCGGTAGATCCCGGC
**RBM47(K317R)**	AAAAAAGCTTATGACCGCAGAGGATTCCACCGC	AAAACTCGAGTCAGTATGTCTGGTAGACGTCGGGG
	GAGGTCACGCTGGCCAGGCCCGTGGACAAGGAG	CTCCTTGTCCACGGGCCTGGCCAGCGTGACCTC
**RBM47(K321R)**	AAAAAAGCTTATGACCGCAGAGGATTCCACCGC	AAAACTCGAGTCAGTATGTCTGGTAGACGTCGGGG
	GCCAAGCCCGTGGACAGGGAGCAGTACTCGCGCT	AGCGCGAGTACTGCTCCCTGTCCACGGGCTTGGC
**RBM47(K329R)**	AAAAAAGCTTATGACCGCAGAGGATTCCACCGC	AAAACTCGAGTCAGTATGTCTGGTAGACGTCGGGG
	TACTCGCGCTACCAGAGGGCAGCCAGGGGCGGC	GCCGCCCCTGGCTGCCCTCTGGTAGCGCGAGTA
**RBM47(K374R)**	AAAAAAGCTTATGACCGCAGAGGATTCCACCGC	AAAACTCGAGTCAGTATGTCTGGTAGACGTCGGGG
	GGACTACTTTGTGAGAGCAGGCAGCATAAGAG	CTCTTATGCTGCCTGCTCTCACAAAGTAGTCC
**RBM47KR(R317K)**	AAAAAAGCTTATGACCGCAGAGGATTCCACCGC	AAAACTCGAGTCAGTATGTCTGGTAGACGTCGGGG
	GTCACGCTGGCCAGGCCCGTG	CACGGGCCTGGCCAGCGTGAC
**RBM47KR(R321K)**	AAAAAAGCTTATGACCGCAGAGGATTCCACCGC	AAAACTCGAGTCAGTATGTCTGGTAGACGTCGGGG
	GTGGACAGGGAGCAGTACTCGCGC	GCGCGAGTACTGCTCCCTGTCCAC
**RBM47KR(R329K)**	AAAAAAGCTTATGACCGCAGAGGATTCCACCGC	AAAACTCGAGTCAGTATGTCTGGTAGACGTCGGGG
	TCGCGCTACCAGAGGGCAGCCAGGGGC	GCCCCTGGCTGCCCTCTGGTAGCGCGA
**RBM47KR(R374K)**	AAAAAAGCTTATGACCGCAGAGGATTCCACCGC	AAAACTCGAGTCAGTATGTCTGGTAGACGTCGGGG
	TACTTTGTGAGAGCAGGCAGCATA	TATGCTGCCTGCTCTCACAAAGTA
**Flag-ISG15**	AAAAGGATCCATGGGCTGGGACCTGACGG	AAAACTCGAGTTAGCTCCGCCCGCCAGG
**ISG15**	AAAAGGATCCATGGGCTGGGACCTGACGG	AAAACTCGAGTTAGCTCCGCCCGCCAGG
**UBE1L**	AAAAAAGCTTATGGATGCCCTGGACGCTTC	AAAACTCGAGTCACAGCTCATAGTGCAGAGGTG
**UBCH8**	AAAAGGATCCATGATGGCGAGCATGCGAGTG	AAAACTCGAGTTAGGAGGGCCGGTCCACTCC
**USP18**	GGAATTCCATATGATGGCCCTCCACAGCCCGCA	AAAAGCGGCCGCTTACAGCAGGTCCACTCGGCGG
**GRK2(K220R)**	AAAAGCTAGCATGGCGGACCTGGAGG	AAAAAAGCTTTCAGAGGCCGTTGGCAC
	AAGATGTACGCCATGAGGTGC	GCTTTTTGTCCAGGCACCTCAT

**HA-HERC5**	AAAAGGATCCATGGAGCGGAGGTCGCGG	AAAACTCGAGTCAGCCAAATCCTCTGTTGTTGTTG
**HECT**	AAAAACCGGTATGTTTGATCTAACAGTC	AAAAGCGGCCGCTCAGCCAAATCCTCTGTT
**SPOP**	GACTACCGGTAGCGTGAACATCTCCGGCCAG	TTCTGCGGCCGCTCAGCTCTGTTTCAGTCTCTTCCTGG
**FLAG-nbRBM47-SPOP**	AAAAGGATCCCAGGTGCAGCTGCAGGAGTCT	AAAAACCGGTTGAGGAGACGGTGACCTG
**FLAG-bv025-SPOP**	AAAAGGATCCCAGGTGCAGCTGCAGGAGTCT	AAAAACCGGTGGAGGACACGGTCACCTG
**FLAG-nbRBM47-HECT**	AAAAGGATCCCAGGTGCAGCTGCAGGAGTCT	AAAAACCGGTTGAGGAGACGGTGACCTG
**FLAG-bv025-HECT**	AAAAGGATCCCAGGTGCAGCTGCAGGAGTCT	AAAAACCGGTGGAGGACACGGTCACCTG
**HA-nbRBM47-HECT**	AAAAAAGCTTCAGGTGCAGCTGCAGGAGTCT	AAAACTCGAGTCAGCCAAATCCTCTGTT
**HA-BV025-HECT**	AAAAAAGCTTCAGGTGCAGCTGCAGGAGTCT	AAAACTCGAGTCAGCCAAATCCTCTGTT
**FLAG-nbRBM47-HECT (C994A)**	AAAAGGATCCCAGGTGCAGCTGCAGGAGTCT<br>CTATAAGAGCACTGACAGCCTTCAGTGTC	AAAAACCGGTTGAGGAGACGGTGACCTG<br>GAGGACACTGAAGGCTGT

Table S3 ALI scoring system

Indicators	Standard	Score
Alveolar septal thickening	no thickening<br>thickened area <5% of the whole lobe<br>thickened area ≤10% of the whole lobe<br>thickened area 10%-25% of the whole lobe<br>thickened area 25%-50% of the whole lobe<br>thickened area >50% of the whole lobe	0<br>1<br>2<br>3<br>4<br>5
Alveolar pattern collapse	no collapse<br>collapsed area <5% of the whole lobe<br>collapsed area ≤10% of the whole lobe<br>collapsed area 10%-25% of the whole lobe<br>collapsed area 25%-50% of the whole lobe<br>collapsed area >50% of the whole lobe	0<br>1<br>2<br>3<br>4<br>5
Inflammatory cell infiltration	no inflammatory cell infiltration<br>infiltration area <5% of the whole lobe<br>infiltration area ≤10% of the whole lobe<br>infiltration area 10%-25% of the whole lobe<br>infiltration area 25%-50% of the whole lobe<br>infiltration area >50% of the whole lobe	0<br>1<br>2<br>3<br>4<br>5
Intra-alveolar exudate and pulmonary hemorrhage	no exudate and pulmonary hemorrhage<br>infiltration area <5% of the whole lobe<br>infiltration area ≤10% of the whole lobe<br>infiltration area 10%-25% of the whole lobe<br>infiltration area 25%-50% of the whole lobe<br>infiltration area >50% of the whole lobe	0<br>1<br>2<br>3<br>4<br>5

*The final score is the sum of the scores divided by the rating items

Table S4 siRNA-Primers for Genes

Gene ID No	SenseSeq (5'–3')	
**ISG15(9636)**	#1GCAACGAAUUCCAGGUGUCTT	#2GAGCACCGUGUUCAUGAAUTT
**Herc5(51191)**	#1GGAAGGAAAUUUCCCUCAATT	#2CCUGAAAGUUGGAAUGAAATT
**UBE1L(7318)**	#1CAUCUUUGCUAGUAAUCUATT	#2GCUCAAAGUCUUUGCCCUATT
**RBM47(54502)**	#1CACGGUGGCUCCAAACGUUCATT	#2GAGGAGAUUGCCAAGGUCATT
**UBE2L6(9246)**	#1GCGAUGAUGCCAAUGUCCUTT	#2GGAGCUGUUCAGAAAGAAUTT
**GRK2(156)**	#1AGCGAUAAGUUCACACGGUUUTT	#2GCGGUGGGAAACAGUUCAUTT
**NC**	#1UUCUCCGAACGUGUCACGUTT	

Table S5 QRT-Primers for Genes

Gene ID No	Forward primer (5'–3')	Reverse primer (5'–3')
**TSC22D3 (1831)**	ATCTGCAACCGCAACATCGACC	GCATACATCAGATGATTCTTCACC
**Rbm47 (245945)**	CCTACAACGCTCTCATCGGGC	GCGGAATATCCTCCGAGGTAGG
**Gapdh (14433)**	CATCACTGCCACCCAGAAGACTG	ATGCCAGTGAGCTTCCCGTTCAG
**IFN-γ (15978)**	CAGCAACAGCAAGGCGAAAAAGG	TTTCCGCTTCCTGAGGCTGGAT
**Cxcl9 (17329)**	CCTAGTGATAAGGAATGCACGATG	CTAGGCAGGTTTGATCTCCGTTC
**Ccl5 (20304)**	CCTGCTGCTTTGCCTACCTCTC	ACACACTTGGCGGTTCCTTCGA
**IL-1β (16176)**	TGGACCTTCCAGGATGAGGACA	GTTCATCTCGGAGCCTGTAGTG
**IL-6 (16193)**	TACCACTTCACAAGTCGGAGGC	CTGCAAGTGCATCATCGTTGTTC
**IL-10 (16153)**	CGGGAAGACAATAACTGCACCC	CGGTTAGCAGTATGTTGTCCAGC
**CXCL10 (15945)**	ATCATCCCTGCGAGCCTATCCT	GACCTTTTTTGGCTAAACGCTTTC
**Blys (24099)**	CTACCGAGGTTCAGCAACACCA	GAAAGCGCGTCTGTTCCTGTGG
**CCL2 (20296)**	GCTACAAGAGGATCACCAGCAG	GTCTGGACCCATTCCTTCTTGG
**GM-CSF (12981)**	AACCTCCTGGATGACATGCCTG	AAATTGCCCCGTAGACCCTGCT
**TNF-α (21926)**	GGTGCCTATGTCTCAGCCTCTT	GCCATAGAACTGATGAGAGGGAG
**IFN-α1 (15962)**	GGATGTGACCTTCCTCAGACTC	ACCTTCTCCTGCGGGAATCCAA
**IFN-β (15977)**	GCCTTTGCCATCCAAGAGATGC	ACACTGTCTGCTGGTGGAGTTC

***QRT-Primers are provided by https://www.origene.com/**

Figure Legends for Supplementary Figures

Supplemental Figure.1 RBM47 is ISGylated at Lys329

(S1A) Detection of RBM47 Protein Expression in four different Human Cancer Cell Lines: A549 (lung cancer), HCT116 (colorectal cancer), CAL27 (tongue cancer), and HepG2 (liver cancer) followed by WB analysis using antibodies recognized RBM47 and Tubulin.

(S1B) Immunoprecipitation (IP) coupled with mass spectrometry revealed that RBM47 interacted with ubiquitin proteasomal system (UPS) proteins. FLAG-RBM47 was expressed in 293T cells for 48h, followed by IP with anti-FLAG magnetic beads subjected to mass spectrometry (IP-MS). Protein profile data for RBM47 interaction were shown. P indicated number of peptides identified by IP-MS, and UP indicated number of unique peptides (S1B upper panel). Protein from Ubiquitin-Proteasome System Interacting with RBM47 as Identified in the BioGRID Database (S1B lower panel).

(S1C) LPS induced the ISGylation of RBM47. A549 cells were treated with 100 ng/ml LPS for 3 or 6 h followed by WB analysis using antibodies recognized RBM47 and Tubulin.

(S1D) A549 cells were treated with different concentrations of LPS (0, 50, 100 ng/ml) for 6 h followed by WB analysis using antibodies recognized RBM47 and Tubulin.

(S1E, S1F) Depletion of UBE1L (S1E) and UBCH8 (S1F) inhibited ISGylation of RBM47.

UBE1L siRNA, UBCH8 siRNA and scrambled siRNA were transfected into A549 cells for 72 hours, followed by WB analysis using antibodies as indicated.

(S1G) USP18 inhibited the ISGylation of RBM47. 293T cells were co-transfected with plasmids expressing the ISGylation modification enzymes including UBE1L, UBCH8, HERC5 and FLAG-ISG15 and RBM47, plus plasmid expressing USP18 as indicated. 48 hours after transfection, cell lysates were subjected to IP using anti-FLAG magnetic beads followed by WB analysis using antibody recognized RBM47. And the cell lysates were analyzed by WB with indicated antibodies.

Supplemental Figure.2 Deficiency of RBM47-ISGylation induces multifaceted immunosuppression in the lung

(S2A) Comparison of the amino acid sequence flanking K329 of human and mouse RBM47. K332 of mouse RBM47 and R332 of human RBM47 were indicated by dotted box.

(S2B) K329R and K332R inhibited the ISGylation of mouse RBM47. 293T cells were co-transfected with plasmids expressing ISGylation modification enzymes including UBE1L, UBCH8, HERC5 and FLAG-ISG15 and plasmids expressing RBM47, K329R, K332R and K329R;K332R mutants for 48 h. The cell lysates were subjected to IP with anti-FLAG magnetic beads followed by WB analysis using antibody recognized RBM47. And cell lysates were subjected to WB analysis with indicated antibodies. The RBM47 slower-migrating band, indicated by open black arrow, were decreased with K332R mutation. The RBM47 slower-migrating band, indicated by red arrow, were decreased with K329R mutation. These two slower-migrating bands were decreased with K329R;K332R mutation.

(S2C) Generation of RBM47 *K329R;K332R* knock-in mice by using CRISPR/Cas-mediated genome engineering. RBM47 sequence flanking 329 of a representative *K329R;K332R* mouse with K329R (AAG to CGC) and K332R (AAG to CGC) substitution. The mutation sites in the donor oligonucleotide was introduced into exon 5 by homologous targeted repair.

(S2D) LPS induced ISGylation of RBM47 in the lungs of WT and *R/WT* mice, but not *R/R* mice. Mice were injected intraperitoneally with LPS (1 mg/kg or 2 mg/kg) or vehicle (saline). 24 hours later, lung tissues were collected and followed by WB using antibodies recognized RBM47 and Tubulin.

(S2E) K329R KI exacerbated the LPS-induced acute lung injury (ALI). The mice were exposed to nebulized LPS at a dose of 3 mg/mL with a final volume of 5 mL or vehicle (saline). After 24 hours, the lung tissues were analyzed using hematoxylin and eosin (H&E) staining. Representative images of H&E-stained lung tissue sections (n=5) were obtained. Scale bars, 100 μm

(S2F-S2I) Expression of basal and LPS-induced chemokines or chemokine receptors were examined by real-time quantitative reverse transcription PCR (RT-qPCR) on lung RNA, isolated from *R/R*, *R/WT* and WT mice. All samples were normalized using GAPDH housekeeping gene expression. Data are presented as mean ± SEM Statistical significance was determined by two-way mean ANOVA (n=8 for each genotype; *, p-value <0.05, **, p-value <0.01, ***, p-value <0.001, ****, P<0.0001). mRNA expression of *CCR2* (S2F), *CCL5* (S2G), *CXCL9* (S2H), *CXCL10* (S2I) were checked.

(S2J-S2L) The experiments were done as in (S2F) except that mRNA expression of cytokines *TNF-α* (S2J), *IL6* (S2K) and *IL10* (S2L) were checked.

(S2M) IFN-γ induced RBM47-ISGylation. Mice were subcutaneous injected with IFN-γ at a dose of 0.3 μg/kg, 1 μg/kg, and 3 μg/kg for 24 hours and lung tissues were collected, followed by WB analysis with antibodies against RBM47, IFN-γ, and Tubulin.

(S2N) Knockdown of IFN-γ decreased RBM47-ISGylation. IFN-γ siRNA and scrambled siRNA were transfected into A549 cells for 72 hours, followed by treatment with LPS (100 ng/ml) for 6 hours, then the cell lysates were subjected to WB analysis with antibodies against RBM47, IFN-γ, and Tubulin.

(S2O, S2P) The experiments were done as in (S2F) except that mRNA expression of cytokines *CCL2* (S2O), *GM-CSF* (S2P) were checked.

Supplemental Figure 3. Deficiency of RBM47-ISGylation promotes lung tumorigenesis

(S3A) The impact of RBM47-ISGylation deficiency on the growth of Lewis lung cancer cells (LLCs) was evaluated by injecting 2.5×10^5 cells into the tail vein of C57BL/6 mice (n=6; age, 8 weeks) for 3 weeks. The incidence of lung tumors was assessed using a statistical graph.

(S3B) Photographs of lung tumors from experiment (S3A).

(S3C) The weight of lung tumors and adjacent tissues observed in mice from experiment (S3A). Statistical significance was determined by one-way mean ANOVA (*, p-value < 0.05, **, p-value < 0.01, ***, p-value < 0.001).

(S3D) Representative images of lung cancer nodules in H&E-stained lungs of urethane treated mice. *R/R* and WT mice (n = 8 for each genotype) received consecutive 10 weekly intraperitoneal urethane injections (1 g/kg), and 8 weeks after urethane injection lung tissues were collected for H&E staining. Scale bars = 100 μm.

Supplemental Figure 4. Deficiency of RBM47-ISGylation mediates immunosuppression by upregulating TSC22D3 mRNA expression

(S4A) Knockdown of RBM47 increased the mRNA expression level of *TSC22D3* in A549 cells. A549 cells were transfected with RBM47 siRNA or scrambled siRNA for 72 hours, total RNA were extracted and RBM47, *TSC22D3* mRNA expression were analyzed by real-time quantitative reverse transcription PCR (RT-qPCR) and normalized to Actb levels. The results of three repeated experiments were statistically analyzed. Data were shown as mean ± SEM. Statistical significance was determined by T-test. (Three replicate tests; *, p-value <0.05, **, p-value <0.01).

(S4B) Overexpression of RBM47 decreased the mRNA level of *TSC22D3* in A549 cells. A549 cells were mock-transfected or transfected with pcDNA3.1 plasmid expressing RBM47 for 48 hours, total RNA were extracted and RBM47, *TSC22D3* mRNA expression were analyzed by quantitative reverse transcription PCR (RT-qPCR) and normalized to Actb levels. The results of three independent repeated experiments were statistically analyzed. Data are presented as mean ± SEM. Statistical significance was determined by T-test. (Three replicate tests *, p-value <0.05, **, p-value <0.01)

(S4C) RBM47 interacted with TSC22D3 transcripts. Cell extracts were purified from A549 cells expressing RBM47 for 48h and immunoprecipitated with control IgG or anti-RBM47 antibodies. Levels of TSC22D3 and Actin transcript, were determined by RT-PCR analysis in RBM47 or IgG-immune complexes.

(S4D) RNA EMSA was performed by incubating biotin-labeled TSC22D3 3′-UTR with His-fused RBM47. Unlabeled TSC22D3-WT and TSC22D3-Mut were used as cold probes for competition. Labeled as shift represents RNA-protein complexes.

(S4E) TSC22D3 expression in the lungs of *R/R* and WT mice were analyzed by immunohistochemistry (IHC).

(S4F) WB analysis of TSC22D3 expression in lungs of urethane treated mice by using antibodies recognized TSC22D3 and Tubulin. Lung tissues were collected from *R/R* and WT mice received

consecutive 10 weekly intraperitoneal urethane injections (1 g/kg, once a week), and 12 weeks after urethane injection.

(S4G) WB analysis of TSC22D3 expression in Lewis xenografts and adjacent tissues by using antibodies recognized TSC22D3 and Tubulin.

(S4H) Adeno-associated virus (AAV5) - mediated shRNA delivery decreased the expression of TSC22D3 in lungs of mice. WT and *R/R* mice received intratracheal injection of AAV5-shTSC22D3 and control AAV5-shNC (2×10^{11} vg) for 6 weeks, followed by intraperitoneal injection saline. The lung tissues were harvested for WB analysis with antibodies recognized TSC22D3 and Tubulin.

(S4I, S4J) Adeno-associated virus (AAV5)-mediated shRNA delivery decreased the expression of TSC22D3 in lungs of mice treated with LPS. WT, *R/WT* and *R/R* mice received intratracheal injection of AAV5-shTSC22D3 and control AAV5-shNC (2×10^{11} vg) for 6 weeks, followed by injection of LPS (2mg/kg). The lung tissues were collected for WB (S4I) analysis with antibodies against TSC22D3 and Tubulin. TSC22D3 expression in the lungs were analyzed by immunohistochemistry (IHC) (S4J).

Supplemental Figure 5. Chimeric E3 ligase induces human RBM47-ISGylation that represses TSC22D3 expression

(S5A) Design of the chimeric E3 ligase nbRBM47-SPOP for the degradation of RBM47. The substrate-binding MATH domain of the E3 adaptor SPOP (amino acids 1–166) was replaced by FLAG-tagged nbRBM47. Negative nanobody bv025 was used as a control to construct bv025-SPOP.

(S5B) 293T cells were transfected with plasmid expressing RBM47-mCherry, nbRBM47-SPOP and bv025-SPOP as indicated for 48 h, followed by WB analysis by using antibodies against RBM47, Flag and Tubulin.

(S5C) Degradation of RBM47 increased the expression of TSC22D3. A549 cells were transfected with plasmid expressing nbRBM47-SPOP and bv025-SPOP for 48 h, followed by WB analysis by using antibodies against RBM47, TSC22D3 and Tubulin.

Supplemental Figure 6. Epinephrine-mediated S309 phosphorylation primes K329 for ISGylation

(S6A) The p-RBM47 antibody recognized the the overexpressed RBM47 and S309D mutant. HEK-293T cells were transfected with plasmids expressing RBM47, S309A or S309D for 48 hours, followed by WB analysis with antibodies recognzied S309 phophorylated RBM47 (p-RBM47) and Tubulin.

(S6B) Calf Intestinal Alkaline Phosphatase (CIP) treatment was found to reduce S309 phosphorylation levels. To achieve this, A549 cells and HEK-293T cells were collected and subjected to Western blot (WB) analysis. PVDF membranes were incubated with CIP (10 U) and Protease Inhibitor Cocktail (PIC) as indicated. The PVDF membranes were then cut into pieces for different treatments.

(S6C) Depletion of GRK2 inhibited the S309 phosphorylation of RBM47. GRK2 siRNA and scrambled siRNA were transfected into A549 cells for 72 hours, followed by WB analysis using antibodies against S309 phosphorylated RBM47 (p-RBM47) and Tubulin.

(S6D) A549 cells were mock-transfected and transfected with plasmids expressing GRK2 for 48 hours, followed by WB analysis using antibodies as indicated.

(S6E) Effect of GRK2 kinase-dead mutant K220R on RBM47 phosphorylation. 293T cells were

mock-transfected or transfected with plasmids expressing GRK2 and the kinase-dead GRK mutant K220R for 48 hours, followed by WB analysis using antibodies as indicated.

(S6F) Epinephrine induced S309 phosphorylation of RBM47. A549 cells were treatment with epinephrine (80 μM) for 0.5h and 1h, followed by WB analysis using antibodies as indicated.

(S6G) Paroxetine inhibited S309 phosphorylation of RBM47. A549 cells were treatment with paroxetine (5 μM) for 0.5h and 1h, followed by WB analysis using antibodies as indicated

Supplemental Figure 1

S1A

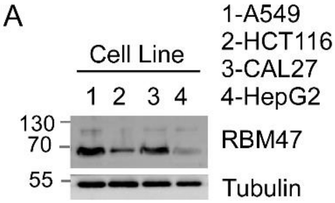

S1C

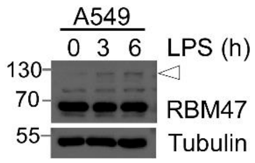

S1D

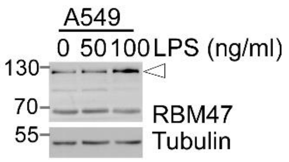

S1B

RBM47 MS

Protein	No.of Unique Peptides	
	P	UP
RBM47	53	46
UBAP2L	4	4
UBA1	19	19
UBA2	1	1
UBA7	13	13
UBAP2L	4	4
UBE2S	1	1
UBE2V2	1	1
UBE2G1	1	1
RNF2	2	1
STUB1	1	1
OTUB1	1	1
UBE2O	1	1
USP5	2	2
USP7	1	1
USP10	1	1

RBM47 bioGRID

Protein
HERC5
RNF2
OTUD4
RPS27A
TRIM11
TRIM25
UBAC2
UBAP2
UBAP2L

S1E

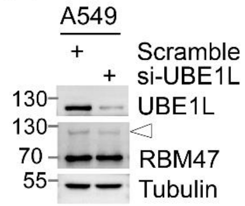

S1F

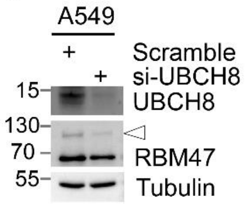

S1G

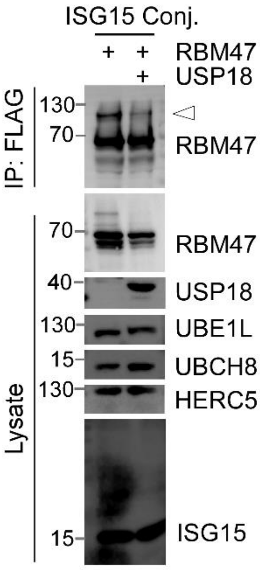

Supplemental Figure 2-1

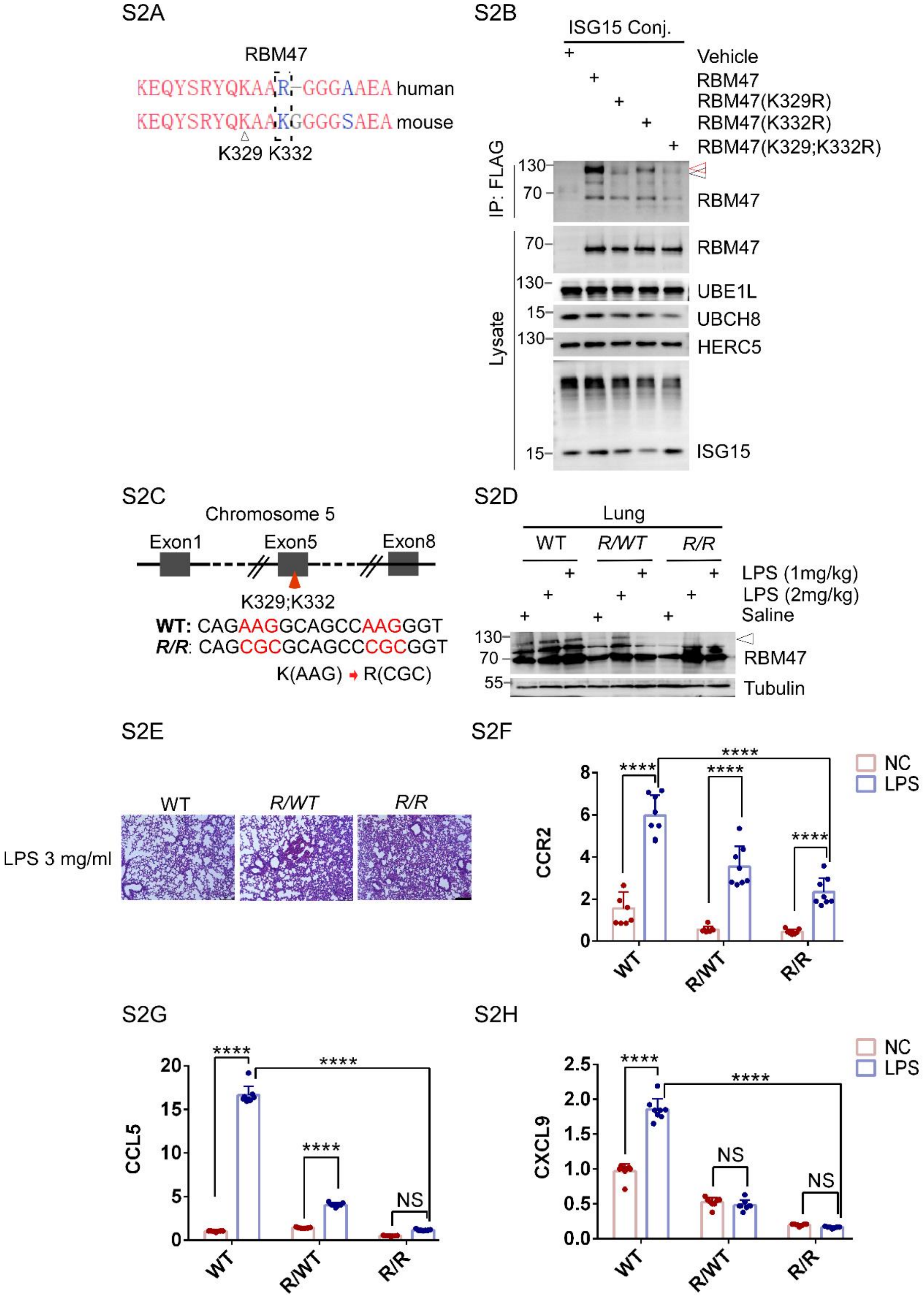

Supplemental Figure 2-2

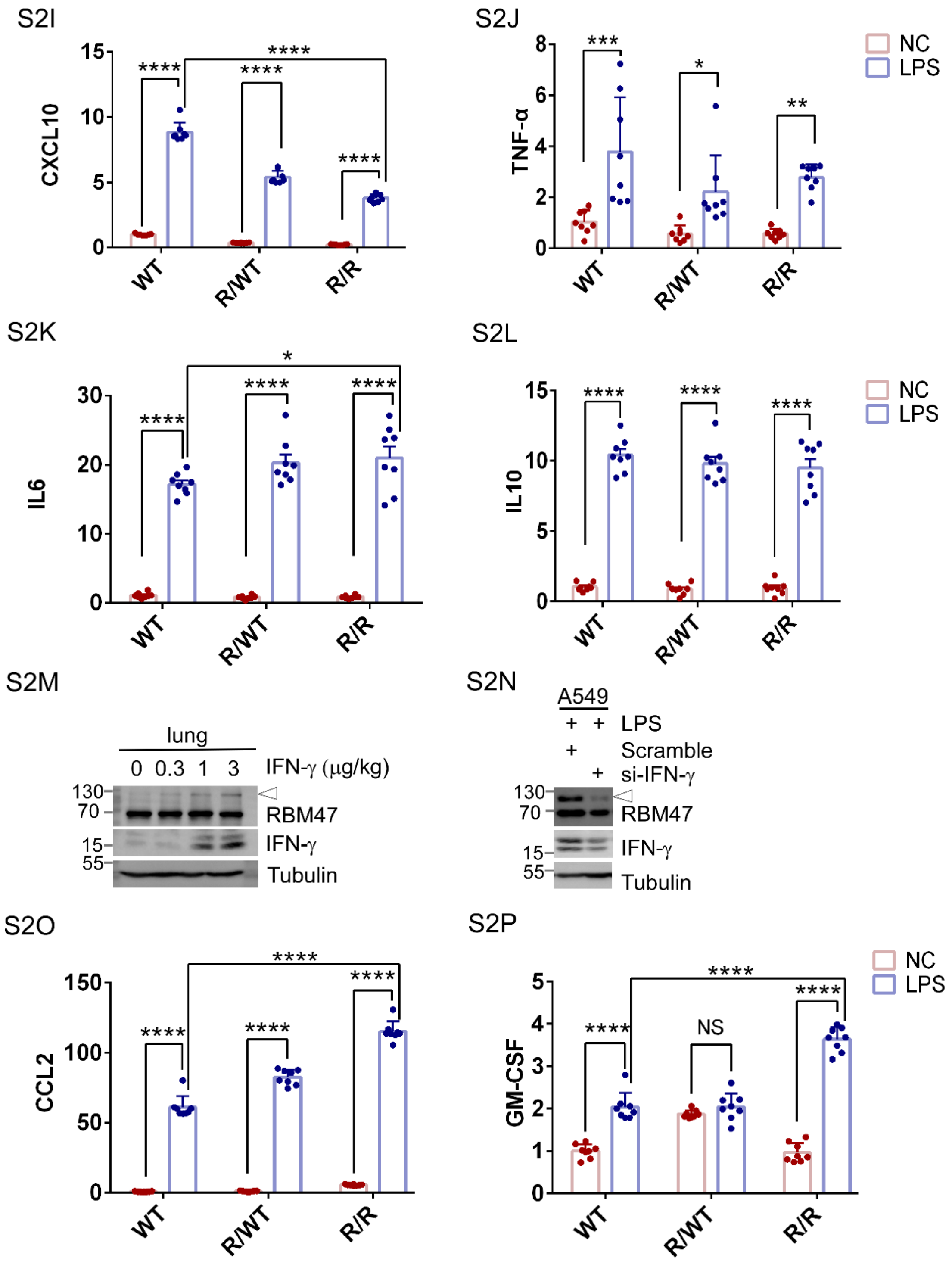

Supplemental Figure 3

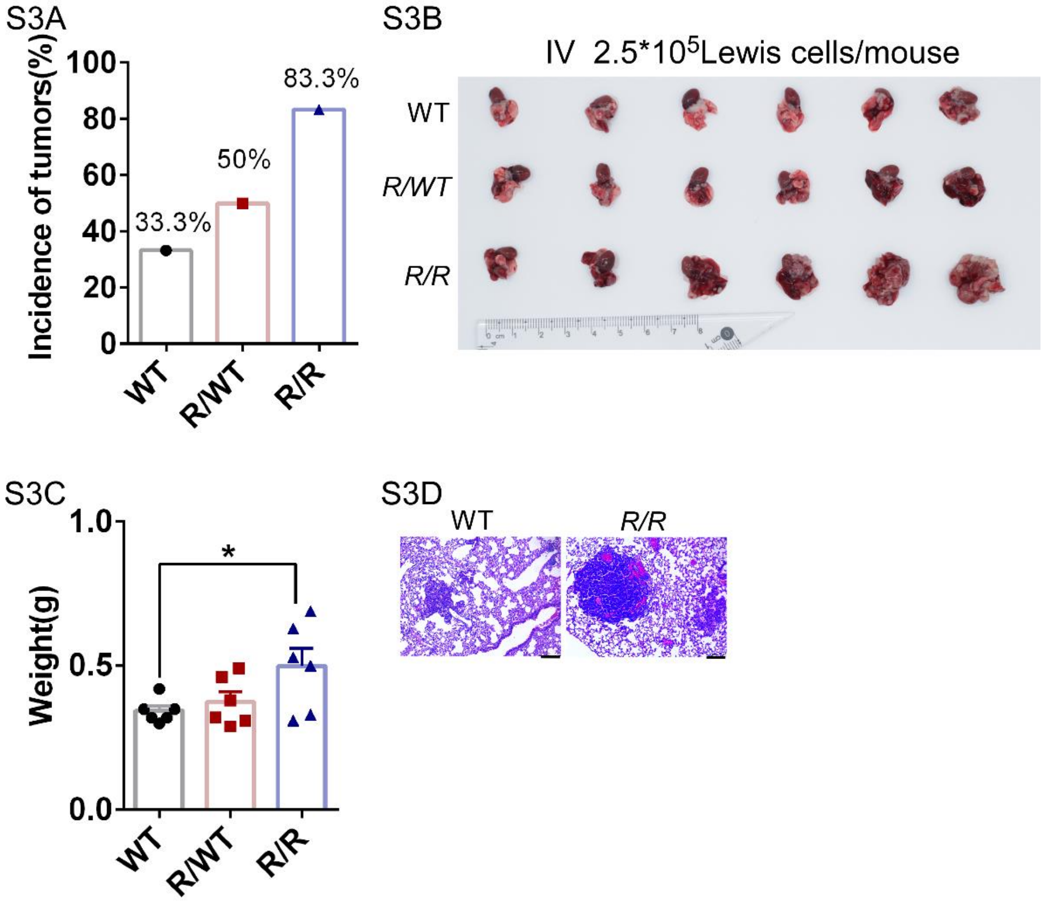

Supplemental Figure 4

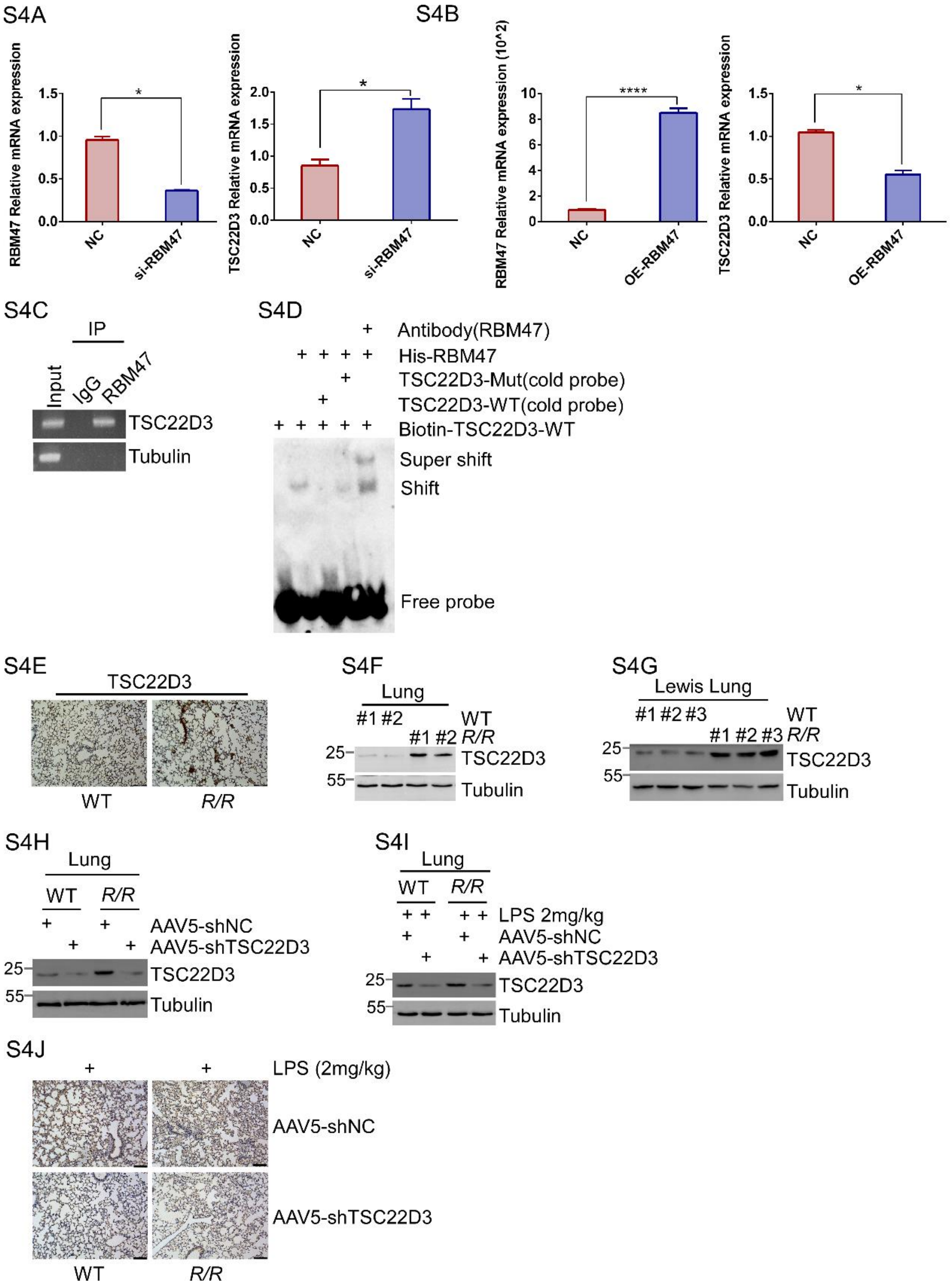

Supplemental Figure 5

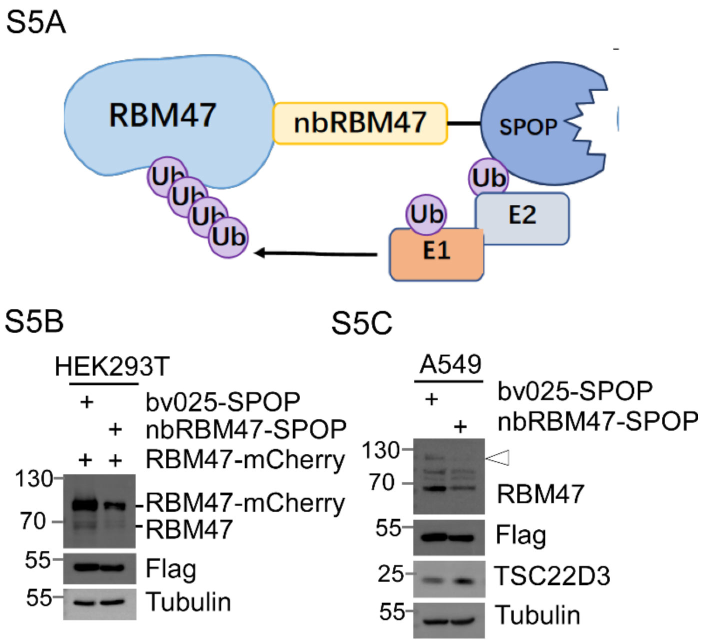

Supplemental Figure 6

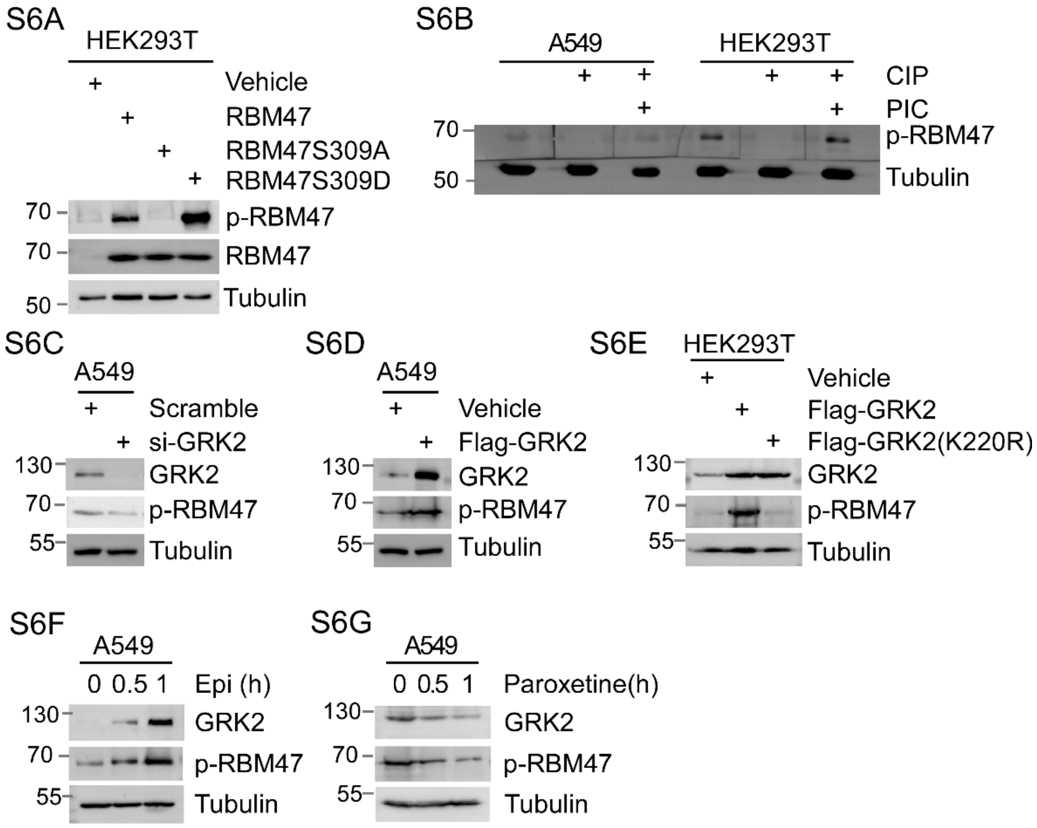

Supplement: Supplementary file 2 — supplemental materials [file 41420_2023_1736_MOESM2_ESM.pdf]
